# Sequence of Two Plasmids from *Clostridium perfringens* Chicken Necrotic Enteritis Isolates and Comparison with *C. perfringens* Conjugative Plasmids

**DOI:** 10.1371/journal.pone.0049753

**Published:** 2012-11-26

**Authors:** Valeria R. Parreira, Marcio Costa, Felix Eikmeyer, Jochen Blom, John F. Prescott

**Affiliations:** 1 Department of Pathobiology, University of Guelph, Guelph, Ontario, Canada; 2 Institute for Genome Research and Systems Biology, Center for Biotechnology, Bielefeld University, Bielefeld, Germany; 3 Bioinformatics Resource Facility, Center for Biotechnology, Bielefeld University, D-33594 Bielefeld, Germany; University of Wisconsin, Food Research Institute, United States of America

## Abstract

Twenty-six isolates of *Clostridium perfringens* of different MLST types from chickens with necrotic enteritis (NE) (15 *netB*-positive) or from healthy chickens (6 *netB*-positive, 5 *netB*-negative) were found to contain 1–4 large plasmids, with most *netB*-positive isolates containing 3 large and variably sized plasmids which were more numerous and larger than plasmids in *netB*-negative isolates. *NetB* and *cpb2* were found on different plasmids consistent with previous studies. The pathogenicity locus NELoc1, which includes *netB*, was largely conserved in these plasmids whereas NeLoc3, present in the *cpb2* containing plasmids, was less well conserved. A *netB*-positive and a *cpb2*-positive plasmid were likely to be conjugative, and the plasmids were completely sequenced. Both plasmids possessed the intact *tcp* conjugative region characteristic of *C. perfringens* conjugative plasmids. Comparative genomic analysis of nine *Cp*CPs, including the two plasmids described here, showed extensive gene rearrangements including pathogenicity locus and accessory gene insertions around rather than within the backbone region. The pattern that emerges from this analysis is that the major toxin-containing regions of the variety of virulence-associated *Cp*CPs are organized as complex pathogenicity loci. How these different but related *Cp*CPs can co-exist in the same host has been an unanswered question. Analysis of the replication-partition region of these plasmids suggests that this region controls plasmid incompatibility, and that *Cp*CPs can be grouped into at least four incompatibility groups.

## Introduction

*Clostridium perfringens* is an important pathogen of humans and animals, and certain strains of type A isolates cause necrotic enteritis (NE) in broiler chickens. NE is a common bacterial infection of chickens that is traditionally controlled by use of antibiotics. However the removal of growth-promoting antibiotics in broiler chickens in Europe and increasing demands elsewhere for “antibiotic-free” chicken are focusing efforts to find alternative approaches to control of this important disease [1], [2]. There has been considerable effort in recent years to understand the pathogenesis of NE in chickens, including understanding the strains involved in disease.

A major breakthrough in understanding virulence in NE isolates of *C. perfringens* was the demonstration that a new toxin, NetB, was critical for development of the disease [3]. Subsequently, our group showed that *netB* formed part of a large pathogenicity locus (PAL) present on a plasmid (NELoc1) and that a second PAL, NELoc3, containing a gene named *hdhA*, which encodes 7-alpha-hydroxysteroid dehydrogenase, was also characteristic of NE isolates and was present on a separate plasmid [4]. A chromosomally encoded locus, NELoc2, was also present in all NE isolates. Pathogenicity loci are clusters of genes that harbour a group of potential virulence genes, which may contribute to the characteristic of the diversity of *C. perfringens* as a pathogen [5], [6].

*Clostridium perfringens* produce at least 15 potent toxins that are responsible for severe diseases in humans and animals [7]. In *C. perfringens*, other than the mouse-lethal alpha toxin genes *cpa* and *cpe*, which may sometimes be chromosomal, the major toxin genes (*cpb*, *etx*, *iap*) currently used to toxinotype *C. perfringens* strains as well as the *netB*, *cpb2* and *tpeL* toxin genes are all harboured on large plasmids [4], [7], [8], [9], [10], [11]. In general, NE strains carry 1 to 4 large plasmids which exhibit considerable diversity in size, ranging from ∼50 to 100 kb [4]. These virulence plasmids share ∼35 kb of conserved backbone sequence which contains among other genes the *tcp* conjugation locus belonging to a family of plasmids referred to as the pCW3-like family [12]. The *tcp* conjugation locus is present on all known conjugative plasmids from *C. perfringens* and consists of 11 genes (*int P*, *tcpA* to *tcpJ*), of which *tcpA*, *tcpF, tcpG, and tcpH* are essential for conjugative transfer [12], [13], [14]. Conjugation systems are important contributors to the dissemination of antibiotic resistance determinants and virulence factors [15]. Recently, three plasmids from an NE isolate have been fully sequenced (pJIR3535, pJIR3844, pJIR3537); one of these contained *netB* and another the *cpb2* gene; the third was a smaller tetracycline-resistance plasmid that did not contain virulence-associated genes [10].

In the study reported here, we sequenced two different plasmids containing NELoc1 and NELoc3 and analysed the diversity of plasmid profiles of a group of *C. perfringens* strains isolated from chickens. Further, comparative genomics tools were used to analyse DNA sequences. We found that both plasmids contained multiple genes which shared high similarity to well-known *C. perfringens* conjugative plasmids (*Cp*CPs).

## Materials and Methods

### Bacterial Strains and Media

Twenty six *C. perfringens* strains belonging to different multi-locus sequence types (ST) were examined (Table 1). NE strains, field isolates from NE cases, and healthy isolates from the same farm in Ontario as the outbreak flock, were obtained from Patrick Boerlin, Department of Pathobiology, University of Guelph [16]. Each isolate was grown overnight at 37°C under anaerobic conditions (80% N_2_, 10% H_2_, 10% CO_2_) on TGY medium (3% Tryptic Soy Broth (Difco Laboratories, Detroit, MI) containing 2% D-glucose (Difco ), 1% yeast extract (Difco), and 0.1% L-cysteine (Sigma-Aldrich Co., St. Louis, MO). All isolates were also cultivated in blood agar (Trypticase Soy Agar (Fisher) with 5% sheep blood) plates aerobically to confirm purity. *E. coli* strains were grown on Luria-Bertani agar plates (Difco) at 37°C in aerobic conditions.

**Table 1:** General features of bacterial strains and plasmids.

Strains/Plasmids	SequenceType^1^	Characteristics/Clinical signs	Source
***E. coli***			
** DH5α**		*F^-^ Φ80 lacZΔM15Δ (lacZYA-argF)U169 endA1 recA1* *hsdr17(r_K_^−^ m_K_^−^ ) deoR thi-1 supE44 gyrA96 relA1*	Invitrogen
***C.perfringens***			
** CW504**		Rif^R2^ Nal^R^ conjugation recipient	J.I.Rood, Monash University
** CP1**		Necrotic enteritis	[34]
**CP1** ***ΔnetB::ErmRAM***		ClosTron insertion in *netB* gene	This study
**CP1** ***Δcpb2::ErmRAM***		ClosTron insertion in *cpb2* gene	This study
** T98**		CW504 derived transconjugant Rif^R^ Nal^R^ Erm^R^with plasmid pCpb2 from CP1*Δcpb2::ErmRAM*	This study
** NE01**	01	Necrotic enteritis	[16]
** NE04**	10	Necrotic enteritis	[16]
** NE06**	02	Necrotic enteritis	[16]
** NE09**	04	Necrotic enteritis	[16]
** NE10**	03	Necrotic enteritis	[16]
** NE14**	05	Necrotic enteritis	[16]
** NE15**	06	Necrotic enteritis	[16]
** NE19**	08	Necrotic enteritis	[16]
** NE20**	09	Necrotic enteritis	[16]
** NE23**	10	Necrotic enteritis	[16]
** NE28**	13	Necrotic enteritis	[16]
** NE30**	14	Necrotic enteritis	[16]
** NE32**	15	Necrotic enteritis	[16]
** NE42**	16	Necrotic enteritis	[16]
** NE57**	22	Necrotic enteritis	[16]
** H+18**	08	Healthy	[16]
** H+22**	01	Healthy	[16]
** H+26**	11	Healthy	[16]
** H+27**	12	Healthy	[16]
** H+34**	10	Healthy	[16]
** H+60**	06	Healthy	[16]
** H-16**	07	Healthy	[16]
** H-45**	17	Healthy	[16]
** H-46**	19	Healthy	[16]
** H-47**	18	Healthy	[16]
** H-54**	20	Healthy	[16]
**Plasmids**			[16]
** pMTL007**		Clostridial vector for expression of ClosTron, containingErmRAM, ColE1, Cm^R^	[19]
** pMTL-netB**		pMTL007 containing intron retargeted to *C. perfringens* *netB*(sense insertion at 461–462 bp)	This study
** pMTL-cpb2**		pMTL007 containing intron retargeted to *C. perfringens* *cpb2*(sense insertion at 390–391 bp)	This study

1 Chalmers *et al*. 2008 [16].

2 Rif^R^, Nal^R^, Cm^R^, Erm^R^, refers to resistance to rifampicin, chloramphenicol and erythromycin, respectively.

### Genomic and Plasmid DNA isolation

Genomic DNA was isolated from 5 ml of overnight culture in Brain Heart Infusion broth (Difco) at 37°C under anaerobic conditions [17]. After precipitation, DNA pellets were washed twice with 70% ethanol and resuspended in TE buffer (10 mM Tris-Cl, pH 7.5 1 mM EDTA). Plasmid DNA was purified using midi-Qiagen columns (Qiagen, Mississauga, Canada) following the manufacturer’s instructions.

### Construction of *netB* and *cpb2* Mutants

The generation of *C. perfringens* mutants was conducted as described in Heap *et al*. [18]. ClosTron intron targeting and design tool (http://clostron.com) identified possible intron target sites. The insertion sites in the sense strands at positions 461/462 bp in the *netB* open reading frame (orf) and 390/391 bp in the *cpb2* orf were preferentially chosen to generate ClosTron-intron modifications, which were obtained by PCR from primers (IBS, EBS2, EBS1 and EBS universal) designed by the ClosTron website (Table S1). The 350-bp products and pMTL007 ClosTron-shuttle vector were both digested with *Hind*III and *Bsr*GI, ligated and then transformed by heat shock into *E. coli* DH5α. Recombinant plasmids were isolated and sequenced in order to verify sequences of the retargeted intron specific for *netB* and *cpb2* insertions. The recombinants pMTLnetB and pMTLcpb2 containing the modified *netB* and *cpb2* intron were then electroporated into electrocompetent *C. perfringens* strain CP1. Recombinant colonies were selected and restreaked onto BHI agar containing 2.5 µg/ml of erythromycin to select for bacteria in which the intron had been inserted. Insertions were confirmed by PCR using the EBS universal primer and target gene specific reverse primers. ErmRAM-F and ErmRAM-R primers were used to demonstrate the ErmRAM splicing, primers used are shown in Table S1.

### Conjugation Experiments

Plasmid transfer experiments were carried out with *C. perfringens* CW504 used as recipient strain. Overnight cultures of *C. perfringens* donor and recipient strains grown in TGY were mixed with a donor:recipient ratio of 2∶1 and a total of 200 µl of both cultures were seeded onto BHI agar without antibiotics and incubated anaerobically at 37°C overnight. Subsequently, the bacterial growth was removed and resuspended in 3 ml of BHI broth. Transconjugants were selected on BHI agar plates supplemented with rifampicin (20 µg/ml), nalidixic acid (20 µg/ml) and erythromycin (2.5 µg/ml). Transconjugants were initially screened by PCR amplifications of specific genes (*netB* and *cpb2*) followed by Pulsed Field Gel Electrophoresis (PFGE) to analyze the presence of plasmids.

### Pulsed Field Gel Electrophoresis

PFGE was performed to analyze the presence of plasmids in 26 poultry *C. perfringens* isolates, as described by Lepp *et al*. [4]. Briefly, DNA plugs for PFGE were prepared from overnight cultures of *C. perfringens* grown in TGY and the bacterial pellets incorporated into a final agarose concentration of 1% in PFGE certified agarose (Bio-Rad Laboratories, Hercules, CA). Plugs were incubated overnight with gentle shaking at 37°C in lysis buffer (0.5M EDTA pH 8.0, 2.5% of 20% sarkosyl (Sigma-Aldrich), 0.25% lysozyme (Sigma-Aldrich) and subsequently incubated in 2% proteinase K (Roche Applied Science, Laval, QC) buffer for 2 days at 55°C. One-third of a plug per isolate was equilibrated in 200 µL of restriction buffer at room temperature for 20 min and then digested with 10 U of *Not*I (New England Biolabs, Pickering, ON) at 37°C overnight. Electrophoresis was performed in a 1% PFGE-certified gel and separated with the CHEF-III PFGE system (Bio-Rad) in 0.56 Tris-borate-EDTA buffer at 14°C at 6 V for 19 h with a ramped pulsed time of 1 to 12 s. Gels were stained in ethidium bromide and visualized by UV light. Mid-Range II PFG markers (New England Biolabs) were used as molecular DNA ladder.

### Preparation of DIG Probes and PFGE Southern Blotting

DNA probes for all PFGE Southern blot steps were labelled by PCR amplification in the presence of digoxigenin-11-dUTP (DIG; Roche Applied Science) according to the manufacturer’s recommendation. DNA probes were amplified from *C. perfringens* strain CP1. DNA probes for *netB* and *hdhA* genes were prepared with specific primers (Table S1). DNA from PFGE gels was transferred to nylon membranes (Roche Applied Science, Mannheim, Germany). DNA hybridizations and detection were performed by using the DIG labelling and CSPD substrate according to the manufacturer’s recommendation (Roche). For Southern blot hybridizations, nylon membranes were prehybridized for at least 2 h at 42°C in hybridization solution without labelled probe and then hybridized separately at 42°C with specific DNA probes for 16 h. The membranes were washed at 68°C under high-stringency conditions. For each different DIG labelled probe, the membrane was first stripped with 0.2 N NaOH and 0.1% sodium dodecyl sulfate, incubated with prehybridization solution, and then reprobed.

### Overlapping PCR Analysis of NE Locus 1–3

A battery of PCR reactions was performed to assess the conservation of NELoc-1–3 among 11 selected poultry isolates. For NELoc-1–3 reactions, a ready-to-use PCR mixture of Platinum PCR SuperMix high-fidelity kit (Invitrogen, Burlington, ON, Canada) was used in a 25 ul reaction containing 0.8 mM of each primer. A touchdown PCR program was used: 94°C for 3 min, 35 cycles of 94°C for 15 s, 65°C to 50°C for 15 s/cycle (the annealing temperature is decreased by 1°C every cycle until 50°C), extension at 68°C for 5 min, and finally, 68°C for 10 min. For longer-range fragments the extension time was increased to 15 min. All primers used are described in Table S1. PCR product sizes were determined by agarose gel electrophoresis and visualized by ethidium bromide staining and photographed under UV light.

### Plasmids Sequencing and Sequence Assemblies

The complete nucleotide sequence of two plasmids from *C. perfringens* pNetB and pCpb2 were determined using the 454 GS Junior Titanium platform (Roche Applied Science, Indianapolis, IN, USA). In brief, plasmid DNA (10 µg) was nebulized at 45 psi for 1 min to shear the DNA into fragments smaller than 400 bp. Sheared DNA was end repaired by incubating with 15 U of T4 polynucleotide kinase and 15 U of T4 DNA polymerase in the presence of buffer and a dNTP mix (10 mM each) at 12°C for 15 min and 25°C for 15 min. DNA was then purified by MinElute PCR Purification Kit (Qiagen). The 454-sequencing adaptors were ligated to the DNA fragments according to the GS Junior Titanium shotgun DNA Library Preparation Method (Roche) by incubating with 104 U of ligase in the presence of ligase buffer at 25°C for 15 min. The reaction was purified by MinElute PCR Purification Kit (Qiagen). The nucleotide sequence reads obtained were assembled using the Newbler (version 2.5p1) *de novo* sequence assembly software (Roche). Gaps between contigs were closed by DNA amplification using conventional PCR techniques and Sanger sequencing.

### Sequencing and Annotation

Complete sequences were automatically annotated by Rapid Annotation using Subsystem Technology (RAST) and manually rectified. BLASTN and BLASTX analyzes were performed to compare the established sequences to known *C. perfringens* plasmids in the NCBI database.

### Comparative Analyses

To analyse the similarity and the phylogeny of the *C. perfringens* conjugative plasmids (*Cp*CPs) the sequences of the sequenced plasmids pNetB-NE10 and pCpb2-CP1 were aligned with sequences of plasmids pCPF5603 (AB236337), pCPF4969 (NC_007772), pJIR3535 (JN689219), pJIR3844 (JN689217), pCPPB-1 (AB604032), pCP8533etx (NC_011412) and pCW3 (NC_010937) using the tool M-GCAT with default settings [19]. A custom PerlScript was used to visualize the alignment mapped on the respective GenBank files [20]. The computation of the core genome (predicted gene products encoded on every plasmid in this study) of the *C.perfringens* plasmids was carried out using the bioinformatics tool EDGAR [21]. The phylogenetic tree was built by the Neighbor-joining algorithm using MEGA5 software [22].

### Nucleotide Sequence Accession Numbers

The *Cp*CP sequences were assigned GenBank accession numbers JQ655731 and JQ655732 for pNetB-NE10 and pCpb2-CP1, respectively.

## Results

### Pulsed-field Gel Electrophoresis and Southern Blot

**Figure 1:**
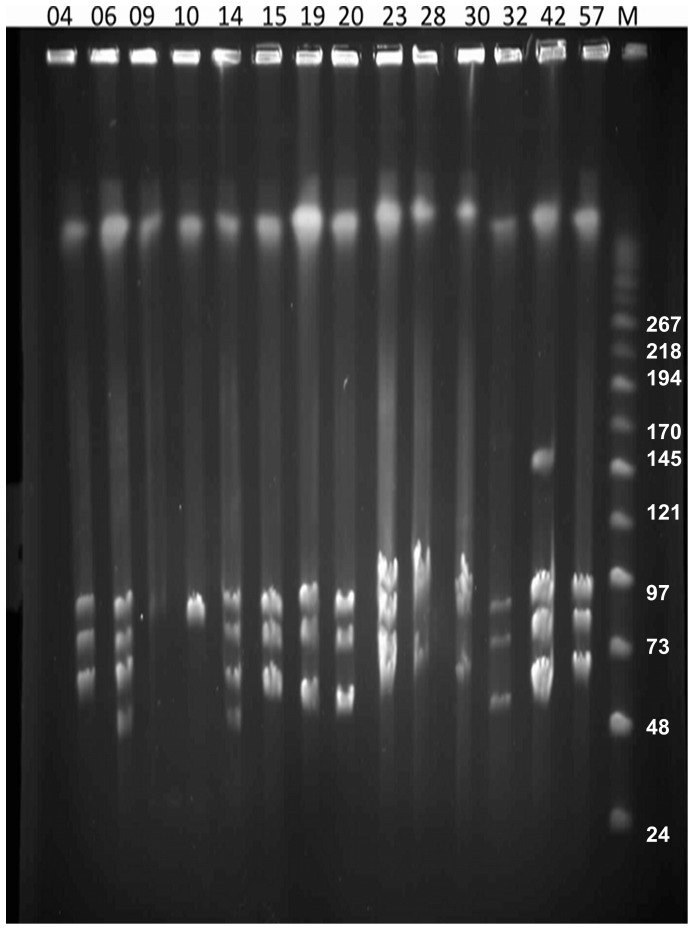
PFGE analyses of plasmids from NE *C. perfringens* poultry strains. Agarose plugs containing DNA from each specified isolate were digested with *Not*I and subjected to PFGE and staining with ethidium bromide. Line numbers indicate isolate numbers M: Mid-Range II PFG molecular DNA ladder (Kb).

**Figure 2:**
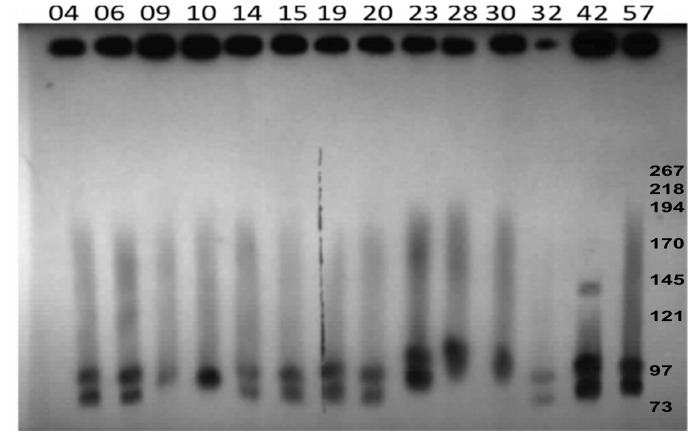
PFGE Southern blot of plasmids from NE *C. perfringens* poultry strains. Southern blotting of PFGE (Figure 1) was performed with DIG-labelled probes for *netB* and *hdhA*. Results from both *netB* and *hdhA* probes are shown overlayed. In all lanes with two bands, the upper band represents *netB* and the lower band *hdhA*. M: Mid-Range II PFG molecular DNA ladder (Kb).

To determine the presence of large plasmids in NE and healthy chicken isolates, DNA from 26 poultry isolates (Table 1) were subjected to PFGE. *In silico* restriction endonuclease analysis of the genomes of *C. perfringens* strains SM101 and ATCC13124, as well as of plasmids pCPF5603, pCPF4969 and pCP8533etx, revealed that *Not*I cleaved the genomes at no more than one location, whereas all plasmids were cleaved once; this restriction enzyme was therefore chosen to linearize the plasmids prior to PFGE. The PFGE profiles of the virulent NE type A strains digested with *Not*I revealed the presence of one to four large plasmids ranging in size from 45 kb–150 kb in all strains (Figure 1, Table 2). PFGE analysis (Figure 1) showed the diversity of plasmids among the type A *C. perfringens* poultry isolates and their sometimes marked size variations, which were confirmed by PFGE/Southern blotting experiments (Figure 2). *C. perfringens* strains NE09 and NE10 carried just a single large *netB*-positive plasmid (Table 2) but most NE isolates carried at least 3 large plasmids, in which the *netB* and *cpb2-hdhA* genes were on distinct plasmids (Figure 2). Interestingly, a group of six *netB*-positive isolates from healthy chickens also showed three to four large plasmids, whereas five *netB*-negative healthy chicken isolates had fewer and smaller large plasmids (Figure S4A).

**Table 2:** Properties of *Clostridium perfringens* strains.

Strains	Plasmids^1^	PCR^3^		Southern blot	
		*netB*	*cpb2*	*netB*	*cpb2*
CP1	4	+	+	90	80
NE01	3	+	+	90	80, 70
NE04	3	+	+	85	75
NE06	4	+	+	85	75
NE09	1	+	−	85	–
NE10	1	+	−	82	–
NE14	4	+	+	88	77
NE15	3	+	+	87	77
NE19	3	+	+	90	77
NE20	3	+	+	87	75
NE23	4	+	+	95	75
NE28	3	+	+	85	80, 70
NE30	3	+	+	85	80, 70
NE32	3	+	+	85	74
NE42	4	+	+	94	81
NE57	3	+	+	93	82
H+18	3	+	+	90	80, 50
H+22	3	+	+	95	85
H+26	3	+	+	95	90, 60
H+27	3	+	+	95	90, 60
H+34	4	+	+	90	80
H+60	3	+	+	90	85
H-16	1	−	+	–	60
H-45	3	−	+	–	65
H-46	3	−	+	–	65
H-47	2	−	+	–	73
H-54	3	−	+	–	60

1 Number of plasmids showed by PFGE analysis. Numbers indicate the approximate size of the plasmid (in kb).

2 Genes detected by PCR amplification. (−) negative;

Southern blotting showed the presence of *cpb2* in two plasmids in the same isolate (Figure S4B). When *Not*I-digested genomic DNA was probed with *netB,* a hallmark of NELoc-1, it hybridized as a single large band in NE isolates as well as in *netB*-positive healthy chicken isolates (Figure S4C). Hybridization to ∼ 80 kb to 100 kb bands confirmed the plasmid identity of these PFGE bands and showed that the *netB* gene was always located in the larger plasmids (Figure 2). The *hdhA* probe (NELoc-3) hybridized to different and smaller plasmids than the *netB*-probe (Table 2, Figure 2), in 13 of 15 virulent NE isolates as well as in all *netB*-positive healthy chicken isolates.

### Mutants and Conjugation

The *netB* and *cpb2* genes were insertionally inactivated in the strain CP1, resulting in the mutant strains CP1Δ*netB*::ErmRAM and CP1Δ*cpb2*::ErmRAM. The insertion of ErmB-carrying introns into the target genes was confirmed by PCR using primers flanking the insertion site (data not shown). The *netB* and *cpb2* genes located in different plasmids in CP1 strain were thus marked with erythromycin-cassette resistance (*ermB*) and this resistance could subsequently be used as a selective marker.

Conjugation assays were performed using *C. perfringens* strains CW504 Rif^R^Nal^R^ as the recipient and CP1Δ*cpb2*::ErmRAM and CP1*ΔnetB*::ErmRAM as donor strains in plate matings. Both plasmids (pNetB and pCpb2) transferred to the recipient strain, however we were unable to find one transconjugant harbouring only pNetB. Erythromycin-resistant transconjugants were confirmed by specific PCR amplifications of *ΔnetB*::ErmRAM *and Δcpb2*::ErmRAM; the *ermB* gene was amplified from the transconjugants but not from the wild-type or donor strains.

### Conservation of NELoc-1, 2 and 3 in Poultry Isolates

Overlapping PCR assays were used to check the diversity of the three loci and their sites of insertion in nine virulent NE strains which represented different ST and plasmid profiles (classified by number and sizes) and two *netB*-positive isolates from healthy chickens (Figure S1). NELoc-1 showed a general uniformity and conservation. For NELoc-2 just one isolate (NE 30) showed no PCR amplification for reaction #5 and two healthy chicken isolates (H26, H34) showed slightly smaller products. Most differences were found in the NELoc-3 (Figure S1).

### Sequencing of Plasmids

**Figure 3:**
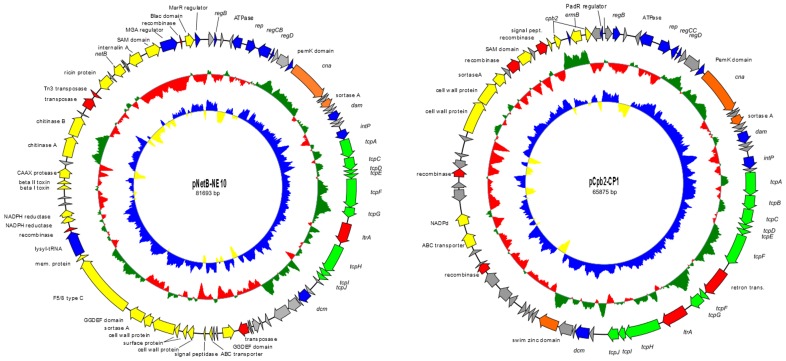
Genetic maps of the sequenced NE plasmids pNetB-NE10 and pCpb2-CP1. The circles represent (from inner to outer most): (i) G + C skew; (ii) G + C content and (iii) open reading frames; arrows indicate the direction of transcription.

The two plasmids pNetB-NE10 and pCpb2-CP1 were isolated from wild-type NE10 and transconjugant T98 (Table 1 and Figure S5) respectively and sequenced on the Roche 454 GS Junior system. The complete nucleotide sequences of the plasmids pNetB-NE10 and pCpb2-CP1 were assembled into circular DNA sequences of 81,693 bp and 65,875 bp with an average depth of coverage of 200, respectively (Figure 3). The average G+C content is 25.7% for pNetB-NE10 and 26.8% for pCpb2-CP1, which is very similar to the G+C content of most *C. perfringens* plasmids [23].

### pNetB-NE10 and pCpb2-CP1

Sequence annotation of pNetB-NE10 showed the presence of 82 open read frames (orfs) whereas pCpb2-CP1 contained 73 orfs (Figure 3). Both plasmids are organized in the typical plasmid backbone of other *C. perfringens* plasmids [10], [11], [24]. Of the fully sequenced *Cp*CPs, the sequences of plasmids pNetB-NE10 and pCpb2-CP1 have identical gene organizations to plasmids pJIR3535 and pJIR3844 [10], respectively. The pNetB-NE10 and pCpb2-CP1 plasmids sequenced in this study are 99.1% and 97.9% similar to previous published plasmids pJIR3535 and pJIR3844, respectively. All these plasmids share a high degree of similarity with a major difference at the orfs 4 and 5 (Table S2).

### Comparative *C. perfringens* Conjugative Plasmid Analyses

**Figure 4:**
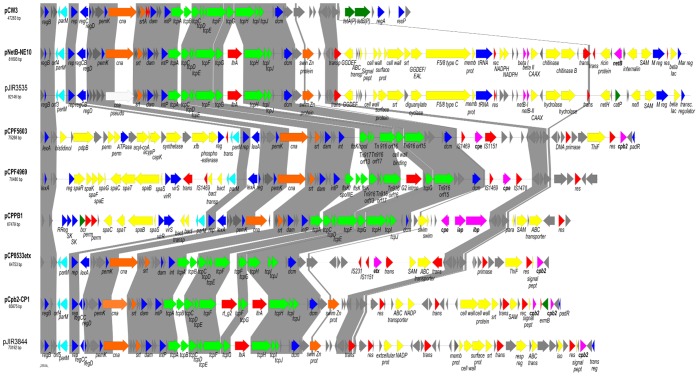
Comparative analysis *of C. perfringens* conjugative plasmids. Comparative analysis of the sequenced NE plasmids pNetB-NE10 and pCpb2-CP1 and the published Cp plasmids pCPF5609, pCPF4969, pJIR3535, pJIR3844, pCPPB1, p8533etx and pCW3. Conserved regions within the analysed plasmids, pNetB (JQ655731), pCpb2 (JQ655732), pCPF5603 (AB236337), pCPF4969 (NC_007772), pJIR3535 (JN689219), pJIR3844 (JN689217), pCPPB-1 (AB604032), pCP8533etx (NC_011412) and pCW3 (NC_010937) are highlighted by grey boxes. Similarities between plasmids were calculated using the M-GCAT tool and visualised using PerlScript.

The DNA sequences of plasmids pNetB-NE10 (JQ655731) and pCpb2-CP1 (JQ655732) were compared to those of plasmids pCPF5603 (AB236337), pCPF4969 (NC_007772), pJIR3535 (JN689219), pJIR3844 (JN689217), pCPPB-1 (AB604032), pCP8533etx (NC_011412) and pCW3 (NC_010937). Figure 4 shows a diagrammatic representation of the organization among these different *Cp*CPs. The software tool EDGAR [21] was used for the assessment of genes that are present on all nine *Cp*CPs and definition of a conserved backbone structure for these plasmids. A total of 24 core genes were identified (Table 3). From 24 core genes, 22 genes belong to the conserved backbone, encoding the plasmid replication protein (*rep*), a DNA-binding transcriptional repressor (*regD*), the PemK protein, a sortase family protein, proteins required for conjugative transfer (*tcpACDEFGHIJ*), a DNA adenine-specific methyltransferase (*dam*), a tyrosine integrase (*intP*) and seven hypothetical proteins, for a total of around 35 kb of the plasmid.

**Table 3:** Core genome genes of *C. perfringens* plasmids.

pNetB-NE10	pCpb2-CP1	Gene/orf	Name/Function
pNetB-NE10_1	pCpb2-CP1_1		hypothetical protein, unknown
pNetB-NE10_6	pCpb2-CP1_6	*rep*	plasmid replication protein
pNetB-NE10_8	pCpb2-CP1_8	*regD*	DNA-binding transcriptional repressor
pNetB-NE10_9	pCpb2-CP1_9		hypothetical protein, unknown
pNetB-NE10_10	pCpb2-CP1_10		hypothetical protein, unknown
pNetB-NE10_11	pCpb2-CP1_11	*pemK*	PemK, growth inhibitor (COG2337)
pNetB-NE10_14	pCpb2-CP1_14		hypothetical protein, unknown
pNetB-NE10_15	pCpb2-CP1_15	*srt*	sortase family protein
pNetB-NE10_16	pCpb2-CP1_16		hypothetical protein, unknown
pNetB-NE10_17	pCpb2-CP1_17		hypothetical protein, unknown
pNetB-NE10_18	pCpb2-CP1_18	*dam*	DNA adenine-specific methyltransferase
pNetB-NE10_19	pCpb2-CP1_19		hypothetical protein, unknown
pNetB-NE10_21	pCpb2-CP1_21	*intP*	tyrosine integrase
pNetB-NE10_22	pCpb2-CP1_23	*tcpA*	conjugation protein TcpA, FtsK/SpoIIIE DNA translocase
pNetB-NE10_23	pCpb2-CP1_25	*tcpC*	conjugation protein TcpC
pNetB-NE10_24	pCpb2-CP1_26	*tcpD*	conjugation protein TcpD
pNetB-NE10_25	pCpb2-CP1_27	*tcpE*	conjugation protein TcpE
pNetB-NE10_26	pCpb2-CP1_28	*tcpF*	conjugation protein TcpF
pNetB-NE10_27	pCpb2-CP1_31	*tcpG*	conjugation protein TcpG
pNetB-NE10_29	pCpb2-CP1_33	*tcpH*	conjugation protein TcpH
pNetB-NE10_30	pCpb2-CP1_34	*tcpI*	conjugation protein TcpI
pNetB-NE10_31	pCpb2-CP1_35	*tcpJ*	conjugation protein TcpJ
pNetB-NE10_40	pCpb2-CP1_53		hypothetical protein, unknown
pNetB-NE10_42	pCpb2-CP1_54		hypothetical protein, unknown

Core genome composed of 24 genes of the nine *C. perfringens* plasmids pCPF4969, pCPF5603, pJIR3844, pCP8533etx, pCPPB-1, pCpb2-CP1, pNetB-NE10, pCW3 and pJIR3535. The core genome was computed with the software tool Edgar.

A second comparative analysis that considered only plasmids from necrotic enteritis isolate*s* showed that 39 common genes among those plasmids (pNetB-NE10, pJIR3535, pCpb2-CP1 and pJIR3844) (Table 4) are conserved. These 39 genes additionally encode the LexA repressor (*regB*), replication protein (*rep*), DNA-binding transcriptional repressor (*regD*), PemK family protein, sortase protein, DNA adenine-specific methyltransferase (*dam*), tyrosine integrase (*intP*), conjugation proteins described above besides conjugation proteins TcpA and TcpI, group II intron reverse transcriptase LtrA, DNA-cytosine methyltransferase (*dcm*), swim zinc finger domain protein and 15 hypothetical proteins of unknown functions in a total of approximately 41 kb of the plasmid size. As expected, the analyzed plasmids differ mostly in genes located in their pathogenicity loci, labelled yellow in Figures 3 and 4.

**Table 4:** Core genome genes of NE *C. perfringens* plasmids.

pNetB-NE10	Gene/orf	Name/Function
pNetB-NE10_1		hypothetical protein, unknown
pNetB-NE10_2	*regB*	SOS-response repressor and protease LexA
pNetB-NE10_6	*rep*	Plasmid replication protein
pNetB-NE10_8	*regD*	DNA-binding transcriptional repressor
pNetB-NE10_9		hypothetical protein
pNetB-NE10_10		cysteine-rich hypothetical protein
pNetB-NE10_11	*pemK*	PemK family protein
pNetB-NE10_13		hypothetical protein
pNetB-NE10_14		hypothetical protein
pNetB-NE10_15	*srt*	Sortase family protein
pNetB-NE10_16		hypothetical protein
pNetB-NE10_17		hypothetical protein
pNetB-NE10_18	*dam*	DNA adenine-specific methyltransferase
pNetB-NE10_19		hypothetical protein, unknown
pNetB-NE10_20		hypothetical protein, unknown
pNetB-NE10_21	*intP*	tyrosine integrase/recombinase
pNetB-NE10_22	*tcpA*	FtsK/SpoIIIE DNA translocase TcpA
pNetB-NE10_23	*tcpC*	conjugation protein TcpC, putative Tn916
pNetB-NE10_24	*tcpD*	conjugation protein TcpD
pNetB-NE10_25	*tcpE*	conjugation protein TcpE
pNetB-NE10_26	*tcpE*	conjugation protein TcpF
pNetB-NE10_27	*tcpF*	conjugation protein TcpG
pNetB-NE10_28	*G2*	group II intron reverse transcriptase LtrA
pNetB-NE10_29	*tcpH*	conjugation pore, membrane protein TcpH
pNetB-NE10_30	*tcpI*	conjugation protein TcpI
pNetB-NE10_31	*tcpJ*	conjugation protein TcpJ
pNetB-NE10_33		hypothetical protein, unknown
pNetB-NE10_34	*dcm*	DNA-cytosine methyltransferase
pNetB-NE10_35		hypothetical protein, unknown
pNetB-NE10_36		hypothetical protein, unknown
pNetB-NE10_37		swim zinc finger domain protein
pNetB-NE10_38		conserved hypothetical protein, unknown
pNetB-NE10_40		conserved hypothetical protein, unknown
pNetB-NE10_42		hypothetical protein, unknown
pNetB-NE10_43		nuclease family transposase
pNetB-NE10_52		cell wall surface anchor family protein
pNetB-NE10_53	*srt*	sortase A, LPXTG specific
pNetB-NE10_58		Recombinase
pNetB-NE10_66		hypothetical protein

Core genome composed of 39 genes of the five NE *C. perfringens* plasmids type A, pJIR3844, pCpb2-CP1, pNetB-NE10, pJIR3535. The core genome was computed with the software tool Edgar.

### Plasmid Central Control Region

**Figure 5:**
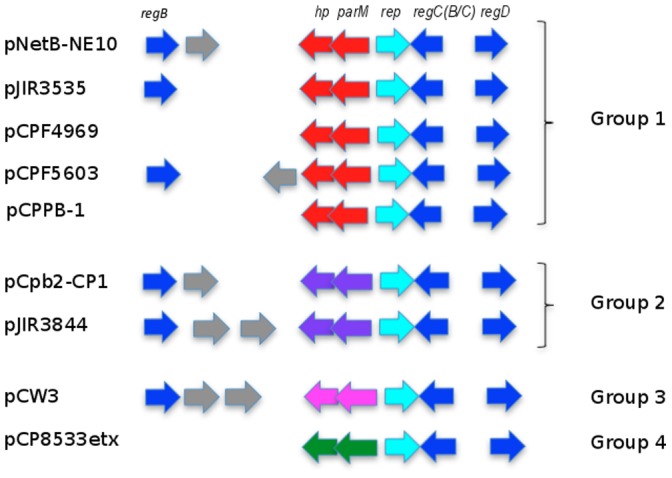
Comparative analysis of central control region of *C. perfringens* conjugative plasmids. Comparative genomic analysis of the central control region of *C. perfringens* plasmids starting from *regB* regulatory gene. Identical colors designate similar function on pNetB-NE10, pCpb2-CP1, pCPF5609, pCPF4969, pJIR3535, pJIR3844, pCPPB1, pCP8533etx and pCW3.

The initial region (∼6 kb sequence) of plasmids pNetB-NE10 and pCpb2-CP1 as well as the two other fully sequenced and annotated NE plasmids (pJIR3535 and pJIR3844), which harbour five genes for regulation (*regB, regCB* or *regCC, regD*), replication (*rep*) or putative partitioning (*parM*), seems to be the “central control region” (CCR) (Figure 5). All nine *C.perfringens* plasmids compared in this study (Figure 4) carry a replication gene (*rep*) encoding a highly conserved replication initiation protein with about 90% identity on nucleotide sequence (Figure S2). Further genes surrounding the *rep* gene appear to be responsible for regulation of plasmid copy number and function as transcriptional repressors. The orf5 (*parM*) of the sequenced NE plasmids (pNetB-NE10, pCpb2-CP1) is found in the CCR, is transcribed divergently from the *rep* gene, and encodes an ATPase involved in putative plasmid partitioning similar to the protein ParM of the ParMRC plasmid partitioning system.

### Phylogenetic Tree

**Figure 6:**
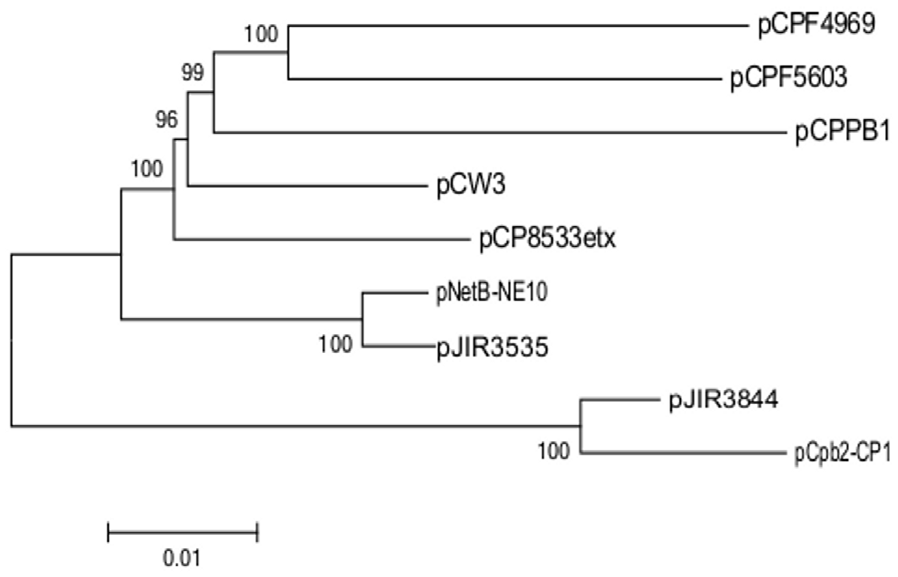
Phylogenetic tree of nine *C. perfringens* conjugative plasmids. The phylogenetic tree was inferred using the Neighbor-joining algorithm [35]. The tree is drawn to scale, with branch lengths in the same units as those of the evolutionary distances used to infer the phylogenetic tree. All positions containing gaps were eliminated from the dataset (Pairwise deletion option). Phylogenetic analyses were conducted in MEGA5.

The sequences of the two sequenced NE plasmids pNetB-NE10 and pCpb2-CP1 and the seven completely sequenced *Cp*CPs were analyzed phylogenetically (Figure 6) as described previously [18]. The phylogenetic tree suggests that these plasmids are closely related phylogenetically, and that there are closer relationships within each of the *netB* and the *cpb2*-containing plasmids. Based on the homologous sequences of all plasmids the % identity varies between 92,3% (pCpb2/pCPF4969) and 99,1% (pNetB/pJIR3535).

## Discussion

The current study provides complete DNA sequences of two NE *C. perfringens* virulence-associated plasmids (pNetB-NE10, pCpb2-CP1), further insight into the conjugative plasmids associated with NE, and significant new understanding of *Clostridium perfringens* conjugative plasmids.

In this study, PFGE analyses revealed the presence of one to four large plasmids >45 kb in fifteen NE isolates of known virulence and different MLST type [16]. The variation in size of the plasmids reported by us here as well as previously [4] suggests that numerous rearrangements occur between and within the large conjugative plasmids, although further plasmid characterization is required to confirm this. For example, Southern blotting showed here for the first time that *cpb2* can be present in two different plasmids within the same host strain (Table 2 and Figure S4). By contrast, in most type A isolates the *netB* gene was found in a single variably sized plasmid (∼80 kb–90 kb). The healthy *netB*-negative chicken isolates lacked the NELoc1 and NELoc3 and their related plasmids, supporting the role of these plasmids in NE.

Overlapping PCR of the three pathogenicity loci [4] confirmed that NELoc-1, which contains *netB*, is very conserved (Figure S1). Size variation of pNetB plasmids therefore must be the result of other changes in these plasmids. In contrast, the NELoc-3 showed greater variation (Figure S1). For example, the isolates NE28, NE30 and H26 possessed just a fragment of the NEloc-3 (*hdhA* gene) and isolate NE42 seemed to harbour only the 5′ and 3′ links of this locus. This suggests that NELoc3, the smallest of the loci associated with NE isolates [4] is less important for NE than the other two loci. The chromosomal NELoc-2 was intact in all except one (NE30) of the eleven strains tested, confirming that it is an important signature for NE isolates. Conjugation assays using the erythromycin resistance-marked NE *C. perfringens* CP1 strain (which contains four large plasmids) as donor and the strain CW504 as recipient resulted in transconjugants with a variable number of the large plasmids (Figure S5), and suggests that all these plasmids are conjugative. Our sequencing data showed that that both pNetB-NE10 and pCpb2-CP1 possess the *tcp* conjugation region, which has been found in all of the conjugative *C. perfringens* plasmids to date [10], [12]. The pNetB-NE10 and pCpb2-CP1 plasmids sequenced in this study are 99.1% and 97.9% similar at the nucleotide level to previous published plasmids pJIR3535 and pJIR3844, respectively (Table S2). The presence of intact *tcp*-based conjugative regions suggests that pNetB-NE10 and pCpb2-CP1 plasmids are conjugative, supporting the recent work of others that showed that pJIR3535 and pJIR3844 plasmids to be conjugative [10].

Analysis of the two new genome sequences of plasmids pNetB-NE10 and pCpb2-CP1 isolated from NE isolates *C. perfringens* (Figure 3) showed the high similarity with two other recently sequenced avian necrotic enteritis *C. perfringens* plasmids pJIR3535 and pJIR3844 [10] and confirmed the extensive conservation of the common backbone among all *Cp*CPs [10], [11], [24], [25], [26].

Comparative genomic analysis showed that *Cp*CPs, including the two plasmids described here, showed greater gene rearrangements including pathogenicity locus and accessory gene insertions around rather than within the backbone region (Figure 4). The *Cp*CPs have a mosaic organization in which transposons and integrases have played a role (Figure 4). The plasmids showed a conserved backbone region highlighted in gray. Hence, differences between the plasmids are related to their pathogenicity locus (yellow), also including different toxin genes (pink) (Figure 4). The nuclease family transposase recognized as orf50 in the pCW3 plasmid seems to be the insertion site of the pathogenicity loci of these conjugative plasmids leading to different organizational types (Figure 4). Inter- and intra-strain rearrangements of *Cp*CPs are apparently responsible for the large size variation of conjugative plasmids from NE *C. perfringens* isolates (Table 2; Figure 1 and 2) possibly by duplications, insertions and deletions. Figure 4 shows a clear pattern of organization around the backbone region and in the pathogenicity loci of the *Cp*CPs, and of the development of these plasmids. The *dcm* region has previously been described as a possible hot spot for insertion of iota-toxin genes in plasmids of *C. perfringens* type E [9], which supports our suggestion of insertional “hot spots” between the regulation and partitioning genes and downstream of the *tcp* transfer region, where *dcm* is located. The pattern that emerges in the analysis shown in Figure 4 is that the major toxin-containing regions of the *Cp*CPs are organized as pathogenicity loci. This was first described for the NELoc1 and NELoc3 of the NE-associated plasmids [4], but might be a general feature of these virulence plasmids.

The comparative analysis of all nine *C. perfringens* plasmids showed a core genome of 24 genes, most of them belonging to the conserved backbone structure (Table 3) which includes the transfer of a clostridial plasmid (*tcp*) locus. The backbone region comprises a large portion of the conjugative plasmids [10], [11], [25], [26], [27], so that for both NE virulence plasmids, as well as for other characterized major virulence plasmids, a size around 35 kb seems to be optimized for efficient replication, conjugative transfer, plasmid maintenance and stability functions. Within the backbone region, there is what we designate the central control region (CCR) consisting of the replication (*rep*), regulatory genes (*reg*) and putative partition genes (*parM*).

The common core genome for NE-isolate associated plasmids is larger (39 genes, around 41 kb). Comparison of the four NE-isolate-associated *C. perfringens* plasmids (pNetB-NE10, pJIR3535, pCpb2-CP1 and pJIR3844) identified not only 35 genes in the backbone region but interestingly also four genes common in the pathogenicity loci. These genes encode a cell wall surface anchor family protein, the sortase A, a resolvase/recombinase and a hypothetical protein. It is clear that *C. perfringens* conjugative plasmids are closely related since they show remarkable homology [8], [11], [27].

Plasmid partition is classified by one of three types of *par* systems. Type I systems use ParA ATPase proteins with Walker-type folds and centromere-binding proteins called ParB; type II systems use actin-like ParM ATPases and centromere-binding proteins called ParR; and a recently described type III system uses a tubulin-like protein, TubZ [28]. The ParMRC operon is a well-known partition system for bacterial DNA segregation in low copy number plasmids [29]. These partition system consists of three components: two genes *parM* and *parR* located side-by-side, with *parM* encoding a NTPase protein, and ParR, a specific centromere-binding protein, and a *cis*-acting centromere-like site *parC*, a small non-coding plasmid region with a series of 11 bp repeats. Interestingly, the equivalent to the ParR protein was not found in any of the *Cp*CPs, but there is a gene adjacent to *parM*, transcribed in the same orientation as *parM*, that encodes a conserved protein of unknown function (orf4 in pNetB-NE10). Sequence differences in the ParM ortholog encoded in the replication and maintenance regions of these plasmids may be involved in this process. The mechanism of segregation is presently unknown for *C. perfringens* conjugative plasmids, and no Par system was described in their DNA sequence.

Six genes were found to be unique within the backbone region for the NE plasmids. These include the collagen adhesion protein (orf12) and a hypothetical protein (orf40) and four genes located in the CCR (orf3, orf4, orf5 *(parM),* orf7 *(regCB),* using pNetB-NE10 as reference). These differences in the CCR transcriptional regulatory genes and segregation genes suggest that these differences may allow this family of plasmids to co-exist in their *C. perfringens* host (Figure 1) and ensure equal inheritance by daughter cells during cell division. There is apparently a limit to the types of large plasmids that a host may carry (Figure 1), which may be a function of the limited variation in the CCR. Gurjar *et al* [27] had earlier suggested that only certain toxin plasmid combinations could be stably maintained within a single *C. perfringens* cell. Earlier analysis suggested that differences in ParM orthologs may be involved in this process [10]. It will be of interest to examine the CCR regions of other *Cp*CPs found in NE strains with multiple plasmids, to determine how these relate to the postulated incompatibility system described here.

The mechanism of the partitioning system incompatibility in *Cp*CPs proposed here is different from the well-known replication-mediated incompatibility [30]. Two different plasmids with the same partitioning system cannot coexist stably in the same host because of the competition between identical partitioning systems [31], [32], [33]. Based on this comparative genomic analysis, we suggest that *C. perfringens* conjugative plasmids can be grouped according to their types of putative partitioning genes, in particular *parM* (*orf5*) and orf4 (hypothetical protein), which seems to be equivalent to *parR* in the ParMRC system.

ATPase/ParM protein showed the highest similarity (99%) in amino acid sequence in the group of plasmids pNetB-NE10 (orf5), pJIR3535 (orf00004) pCPF4969 (orf61), pCPF5603 (orf16) and pCPPB-1 (orf63) (Figure5) (Fig S2). Amino acid sequence alignments showed that the ParM proteins contain conserved domain actin-like ATPases (PRK13917) and a predicted function of a plasmid segregation protein as part of a type II Par system [28], [29]. Plasmids pCpb2-CP1 (orf5) and pJIR3844 (orf6) form a second group that encodes a different ATPase with no conserved domain and just 27% protein identity with orthologues of the first plasmid group. Although ATPase/ParM proteins from plasmids pCW3 (orf13) and pCP8533etx (orf52) have low homology (27%) with each other, both proteins belong to a superfamily of StbA proteins, a family that consists of several bacterial StbA plasmid stability proteins.

The orf4 gene in pNetB-NE10 and its homologues in other *Cp*CPs have no conserved domain or significant similarity to other known proteins in GenBank. Speculatively, this hypothetical protein is suggested to be the potential ParR component of the partitioning system of *Cp*CPs, primarily because it is located adjacent to *parM* with the same transcriptional orientation in all *Cp*CPs analysed. Interestingly, this hypothetical protein is also conserved in the same plasmid groups described above, as is shown in the multiple sequence alignments (Figure S2). Another important element to complete the ParMRC system is the presence of *parC*, the centromeric region of the plasmid. Centromeres consist of a series of tandem DNA repeats of eight 10-bp or four 20-bp repeats typically located adjacent of the *parM* gene [28], [29]. However, the precise size and organization of the *parC* site varies among ParMRC system [29]. The upstream sequence of *parM* genes of the nine *Cp*CPs revealed several imperfect 11 bp repeats and conserved regions among the sequences which appear to be the equivalent of a *parC* site (Figure S3).

In conclusion, the complete sequencing of two new conjugative plasmids from NE isolates described here, when combined with comparative analysis of previously sequenced plasmids, adds considerably to understanding the evolution of virulence-associated plasmids in *C. perfringens*, and contributes to the unanswered question of how these different but related plasmids can co-exist in the same host. The suggestion proposed here of classifying *Cp*CPs into incompatibility groups, of which four are described here, based on the partitioning systems, requires confirmation by experimental data. There are important areas still to be understood including the function of conserved hypothetical proteins, the presence of additional plasmid incompatibility systems, and the basis of any limitation of specific *Cp*CP family members to particular *C. perfringens* types. Sequencing of further large *Cp*CPs (Figure 1) might add confirmation to our supposition about the role of the CCR in maintenance of different family members in the same host.

## Supporting Information

Figure S1 **Overlapping PCR analysis** **of NE locus in C. perfringens.** PCR reactions were performed using DNA from *C. perfringens* strains described on Table 1. Healthy and NE *C. perfringens* isolates H26, H34, NE04, NE09, NE10, NE14, NE20, NE23, NE28, NE30, NE42, respectively. Genetic organization of NE loci. **(A)** Overlapping PCR analysis of NE locus 1. **(B)** Overlapping PCR analysis of NE locus 2. **(C)** Overlapping PCR analysis of NE locus 3. PCR products spanning the entire locus are represented by black bars and the PCR results for each strain tested are given below as follows: +.PCR product was of expected size; −, no PCR product produced. Where the PCR product did not match the expected size, the actual size is given.
(PPT)

Figure S2 **Amino acid alignments of proteins encoded by different** ***C. perfringens*** **plasmids.** Plasmid names and their respective orf number (plasmid name orf#) are described for each protein. Identical residues (*), conservative amino acid substitutions (:), and semi-conservative amino acid substitutions (.) are shown below the aligned sequences. (MUSCLE −3.7).
(DOCX)

Figure S3 **Repeats found on the upstream region of** ***parM*** **gene.** Possible tandem repeats found on the upstream region of *parM* gene next to rep gene from *C. perfringens* plasmids using etandem (http://emboss.bioinformatics.nl/cgi-bin/emboss/etandem).
(DOCX)

Figure S4 **PFGE and Southern blot analyses of plasmids from healthy** ***C. perfringens*** **poultry strains. (A)** PFGE analyses of plasmids from healthy *C. perfringens* poultry strains. Agarose plugs containing DNA from each specified isolate were digested with *Not*I and subjected to PFGE and staining with ethidium bromide. See Table1 and 2 for isolate features. Line numbers indicate isolate numbers M: Mid-Range II PFG molecular DNA ladder (Kb). **(B)** PFGE Southern blot of plasmids from healthy C. perfringens poultry strains. Southern blotting of PFGE (Figure S4A) was performed with only DIG-labelled probe for cpb2 gene. M: Mid-Range II PFG molecular DNA ladder (Kb). **(C)** PFGE Southern blot of plasmids from healthy *C. perfringens* poultry strains. Southern blotting of PFGE (FigureS 4A) was performed with only DIG-labelled probe for *netB* gene. M: Mid-Range II PFG molecular DNA ladder (Kb).
(DOCX)

Figure S5 **PFGE analyses of plasmids from transconjugants** ***C. perfringens*** **strains.** Agarose plugs containing DNA from each specified isolate were digested with *Not*I and subjected to PFGE and staining with ethidium bromide. Lines indicate: CW504 recipient strain (plasmid free); T98 (transconjugant carrying the plasmid pCpb2); T117 (transconjugant carrying three of CP1 plasmids); T118 (transconjugant carrying four of CP1 plasmids); T119 (transconjugant carrying two of CP1 plasmids); T125(transconjugant carrying two of CP1 plasmids); T128 (transconjugant carrying two of CP1 plasmids); CP1 donor strain (harbours four large plasmids); M: Mid-Range II PFG molecular DNA ladder (Kb).
(DOCX)

Table S1 **List of primers. (A)** Primers used for PCR DIG labelling and mutation **(B)** Primers used for overlapping PCR reactions of the three Pathogenicity loci characteristic of necrotic enteritis *C. perfringens* isolates.
(DOCX)

Table S2 **Comparison of NE** ***C. perfringens*** **plasmids. (A)** Comparison of coding sequences pNetB-NE10 and pJIR3535 NE C. *perfringens* plasmids by means of BLASTn analyses. Open reading frames are labeled according to the annotation of plasmid pNetB-NE10 **(B)** Comparison of open reading frames pCpb2-CP1 and pJIR3844 NE *C. perfringens* plasmids by means of BLASTn analyses. Open reading frames are labeled according to the annotation of plasmid pCpb2-CP1.
(DOCX)
