# Supplementary material for: Sequence of Two Plasmids from Clostridium perfringens Chicken Necrotic Enteritis Isolates and Comparison with C. perfringens Conjugative Plasmids
Source: PLoS One. 2012 Nov 26;7(11):e49753. doi: 10.1371/journal.pone.0049753 (PMC3506638; doi:10.1371/journal.pone.0049753)
Supplement: Figure S1 — Overlapping PCR analysis of NE locus in C. perfringens. PCR reactions were performed using DNA from C. perfringens strains described on Table 1. Healthy and NE C. perfringens isolates H26, H34, NE04, NE09, NE10, NE14, NE20, NE23, NE28, NE30, NE42, respectively. Genetic organization of NE loci. (A) Overlapping PCR analysis of NE locus 1. (B) Overlapping PCR analysis of NE locus 2. (C) Overlapping PCR analysis of NE locus 3. PCR products spanning the entire locus are represented by black bars and the PCR results for each strain tested are given below as follows: +.PCR product was of expected size; −, no PCR product produced. Where the PCR product did not match the expected size, the actual size is given. (PPT) [file pone.0049753.s001.ppt]

## Slide 1
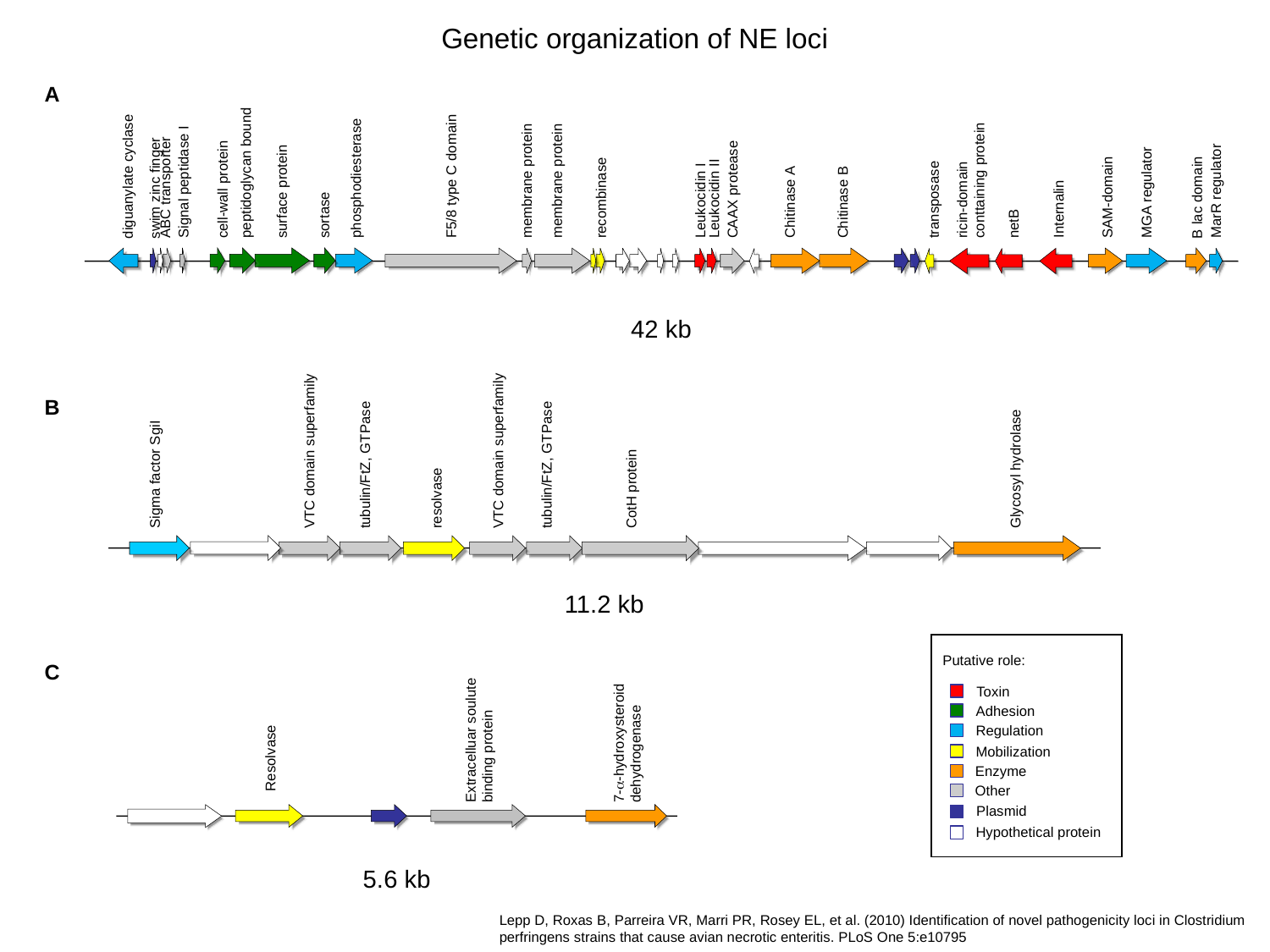

Genetic organization of NE loci
A
ricin-domain
conttaining protein
peptidoglycan bound
diguanylate cyclase
F5/8 type C domain
phosphodiesterase
membrane protein
membrane protein
Signal peptidase I
ABC transporter
swim zinc finger
CAAX protease
cell-wall protein
MarR regulator
surface protein
MGA regulator
Β lac domain
SAM-domain
recombinase
Leukocidin II
transposase
Leukocidin I
Chitinase A
Chitinase B
Internalin
sortase
netB
42 kb
VTC domain superfamily
tubulin/FtZ, GTPase
tubulin/FtZ, GTPase
Sigma factor SgiI
CotH protein
resolvase
B
Glycosyl hydrolase
VTC domain superfamily
11.2 kb
Putative role:
Extracelluar soulute binding protein
7-α-hydroxysteroid dehydrogenase
Resolvase
C
Toxin
Adhesion
Regulation
Mobilization
Enzyme
Other
Plasmid
Hypothetical protein
5.6 kb
Lepp D, Roxas B, Parreira VR, Marri PR, Rosey EL, et al. (2010) Identification of novel pathogenicity loci in Clostridium perfringens strains that cause avian necrotic enteritis. PLoS One 5:e10795

## Slide 2
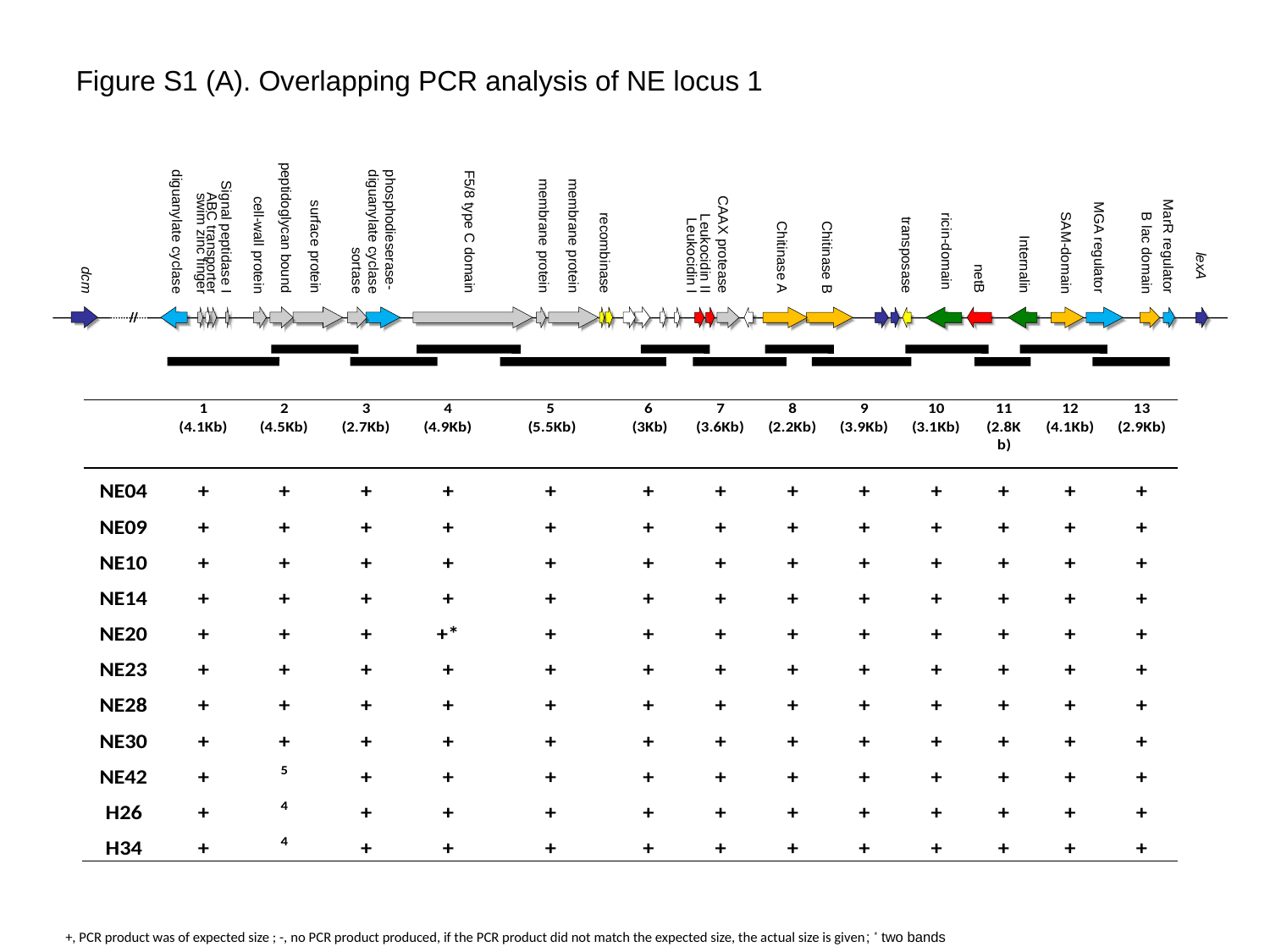

Figure S1 (A). Overlapping PCR analysis of NE locus 1
phosphodieserase-
diguanylate cyclase
peptidoglycan bound
diguanylate cyclase
F5/8 type C domain
membrane protein
membrane protein
Signal peptidase I
ABC transporter
swim zinc finger
CAAX protease
cell-wall protein
MarR regulator
surface protein
MGA regulator
Β lac domain
SAM-domain
recombinase
ricin-domain
Leukocidin II
transposase
Leukocidin I
Chitinase A
Chitinase B
Internalin
sortase
lexA
netB
dcm
+, PCR product was of expected size ; -, no PCR product produced, if the PCR product did not match the expected size, the actual size is given; * two bands

## Slide 3
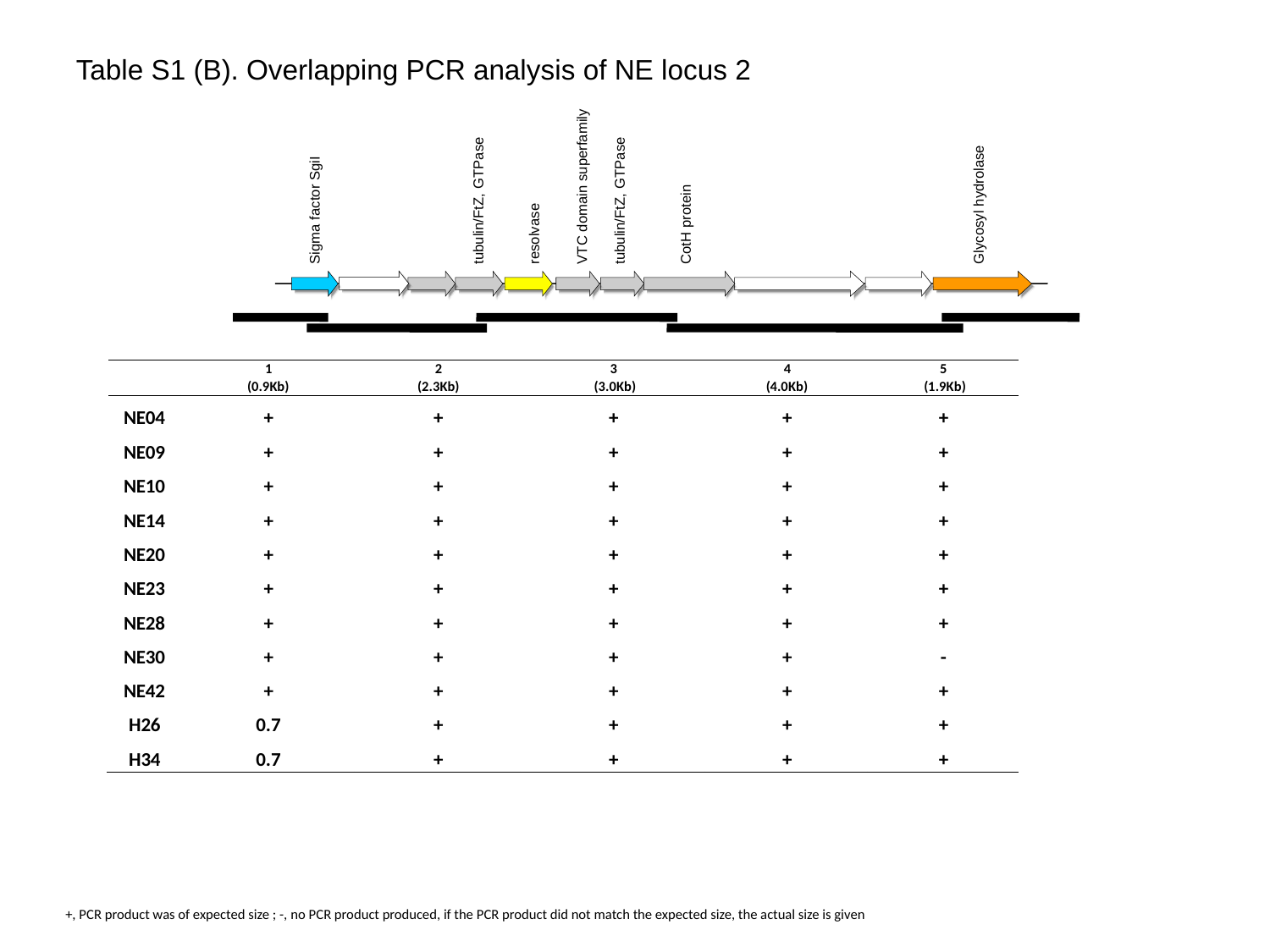

Table S1 (B). Overlapping PCR analysis of NE locus 2
VTC domain superfamily
tubulin/FtZ, GTPase
tubulin/FtZ, GTPase
Sigma factor SgiI
CotH protein
resolvase
Glycosyl hydrolase
+, PCR product was of expected size ; -, no PCR product produced, if the PCR product did not match the expected size, the actual size is given

## Slide 4
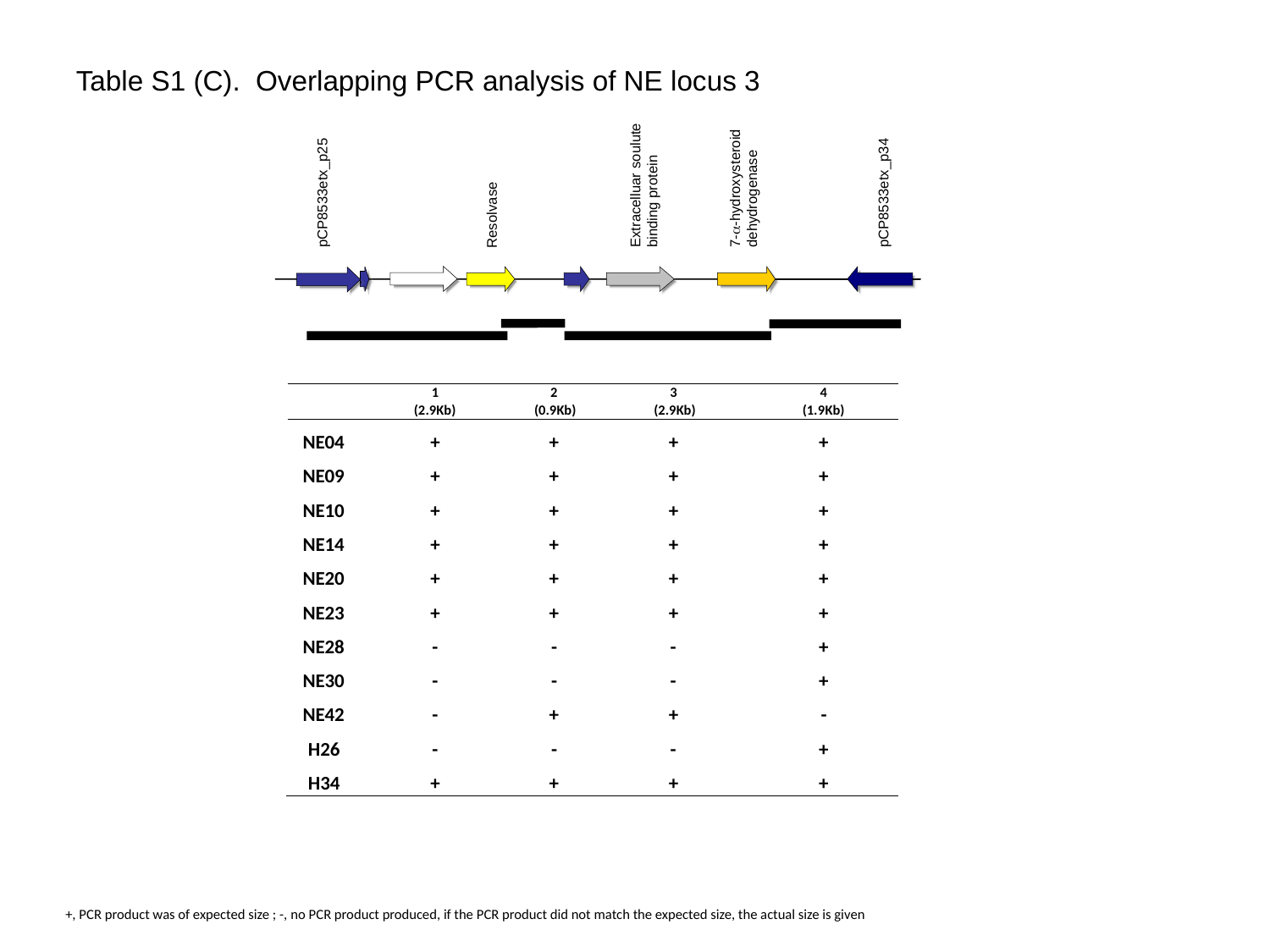

Table S1 (C). Overlapping PCR analysis of NE locus 3
Extracelluar soulute binding protein
7-α-hydroxysteroid dehydrogenase
pCP8533etx_p25
pCP8533etx_p34
Resolvase
+, PCR product was of expected size ; -, no PCR product produced, if the PCR product did not match the expected size, the actual size is given
