# Supplementary material for: Sequence of Two Plasmids from Clostridium perfringens Chicken Necrotic Enteritis Isolates and Comparison with C. perfringens Conjugative Plasmids
Source: PLoS One. 2012 Nov 26;7(11):e49753. doi: 10.1371/journal.pone.0049753 (PMC3506638; doi:10.1371/journal.pone.0049753)
Supplement: Figure S2 — Amino acid alignments of proteins encoded by different C. perfringens plasmids. Plasmid names and their respective orf number (plasmid name orf#) are described for each protein. Identical residues (*), conservative amino acid substitutions (:), and semi-conservative amino acid substitutions (.) are shown below the aligned sequences. (MUSCLE −3.7). (DOCX) [file pone.0049753.s002.docx]

**Figure S2. Amino acid alignments of proteins encoded by different *C. perfringens* plasmids. Plasmid names and their respective orf number (plasmid name_orf#)are described for each protein. Identical residues (*), conservative amino acid substitutions (:), and semi-conservative amino acid substitutions (.) are shown below the aligned sequences. (MUSCLE -3.7)**

**RegB (pNetB-NE10_orf2)**

pJIR3844_00002 MKKREQIYTFLIDFIKEKGYQPTVREIAHAVNLKSSSSVYRHLEMLEKDGLIILGKSEQR

pCW3_0009 MKKREQIYTFLIDFIKEKGYQPTVREIAHAVNLKSSSSVYRHLEMLEKDGLIILGKSEQR

pNetB-NE10_2 MKKREQIYTFLIDFIKEKGYQPTVREIAHAVNLKSSSSVYRHLEMLEKDGLIILGKSEQR

pCPF5603_39 MKKREQIYTFLIDFIKEKGYQPTVREIAHAVNLKSSSSVYRHLEMLEKDGLIILGKSEQR

pCpb2-CP1_2 MKKREQIYTFLIDFIKEKGYQPTVREIAHAVNLKSSSSVYRHLEMLEKDGLIILGKSEQR

pJIR3535_00002 MKKREKIYTFLINFIKEKGYQPTVREIAHAVNLKSSSSVYRHLEMLEKYGLIILGKSEQR

*****:******:*********************************** ***********

pJIR3844_00002 GRKRSIHIIDLNKKIKKNVSEETIKNLIKFSKKQVNTNNDVFPKKVTNDYIVTVDNIFPD

pCW3_0009 GRKRSIHIIDLNKKIKKNVSKETIKNLIKFSKKQVNTNNDIFSKKVTNDYIVAVDNIFPD

pNetB-NE10_2 GRKR--------------------------------------------------------

pCPF5603_39 GRKRSIHIIDLNKKIKKNVSEETIKNLIKFSKKQVNTNNDVFPKKVTNDYIVTVDNILPD

pCpb2-CP1-CP1_2 GRKRSIHIIDLNKK---NVSEETIKNLIKFSKKQVNTNNDVFPKKVTNDYIVTVDNILPD

pJIR3535_00002 SRKR--------------------------------------------------------

.***

pJIR3844_00002 DIIIVEKVNSLEKDSIGLLLFENHTFIKKIFSDYEKVYDTDPVVLGKIIGVYRDFF

pCW3_0009 DIIIVEKVNSLEKDSIGLLLFENRTFIKKIFSDYEKVYDTDPIVLGKIIGVYRDFF

pNetB-NE10_2 --------------SIGLLLF-----------------------------------

pCPF5603_39 DIIIIEKVNSLEKDSIGLLLFENHTFIKKIFSDYEKVYDTDPVVLGKIIGVYRDFF

pCpb2-CP1_2 DIIIIEKVNSLEKDSIGLLLFENHTFIKKIFSDYEKVYDTDPVVLGKIIGVYRDFF

pJIR3535_00002 --------------------------------------------------------

**Hypothetical protein (pNetB-NE10_orf4)**

pCPF5603_17 MANIERVNLRLNLDDPRDAAIWDLVKDKKNKKGTYIKFLIYNLIVGNEISITKNSDKNFA

pJIR3535_00003 MANIERVNLRLNLDDPRDAAIWDLVKDKKNKKGTYIKFLIYNLIIGNEITITKNSDKNFA

pNetB-NE10_4 MANIERVNLRLNLDDPRDAAIWDLVKDKKNKKGTYIKFLIYNLIIGNEITITKNSDKNFA

pCPF4969_60 MANIERVNLRLNLDDPRDAAIWGLIKDKKNKKGTYIKFLVYNLIVGNEISIT----KSFV

pCPPB-1_64 MANIERVNLRLNLDDPRDAAIWDLIKDKKNKKGTYIKFLVYNFIVGNEISIT----KNFV

**********************.*:**************:**:*:****:** *.*.

pCPF5603_17 NSDNDKNDISEDEFDDDINNEFG

pJIR3535_00003 NSENDKNDISEDEFDDDINNEFA

pNetB-NE10_4 NSENDKNDISEDEFDDDINNEFA

pCPF4969_60 NSYSDKNDISEDEFDDDINNEFG

pCPPB-1_64 NSDIDKNDISEDEFDDDINNEFG

** ******************.

**ParM (pNetB-NE10_orf5)**

pCPF4969_61      ----------MEVLKLIIAVDLGNYNIKTCEGIIFESRYQEVEKEDFDTDLLELENKFYR
pCPPB-1_0        SYFVANNFKKMEVLKLIIAVDLGNYNIKTCEGIIFESRYQEVEKEDFDTDLLELENKFYR
pCPPB-1_63       ---------------LIIAVDLGNYNIKTCEGIIFESRYQEVEKEDFDTDLLELENKFYR
pJIR3535_00004 ---------------LIIAVDLGNYNIKTCEGIIFESRYQKVEKEDFDTDLLELENKFYR
pNetB-NE10_5     ---------------LIIAVDLGNYNIKTCEGIIFESRYQKVEKEDFDTDLLELENKFYR
pCPF5603_16      ---------------LIIAVDLGNYNIKTCEGIIFESRYQEVEKEDFDTDLLELENKFYR
                                *************************:*******************

pCPF4969_61      MESGDFENEFNKAKKNFIPNLLYAISKSCDVDNIETDLVLGVPASNLGISEELKEKLLNK
pCPPB-1_0        MESGDFENEFNKAKKNFIPNLLYAISKSCDVDDIEADLVLGVPASNLGISEELKEKLLNK
pCPPB-1_63       MESGDFENEFNKAKKNFIPNLLYAISKSCDVDDIEADLVLGVPASNLGISEELKEKLLNK
pJIR3535_00004 MESGDFENEFNKAKKNFIPNLLYAISKSCDVDNIETDLVLGVPASNLGISEELKEKLLNK
pNetB-NE10_5     MESGDFENEFNKAKKNFIPNLLYAISKSCDVDNIETDLVLGVPASNLGISEELKEKLLNK
pCPF5603_16      MESGDFENEFNKAKKNFIPNLLYAISKSCDVDNIETDLVLGVPASNLGISEELKEKLLNK
                 ********************************:**:************************

pCPF4969_61      CFNFSLFEVNKNIKINKVATVAEGLSSFYTLPKEERAKDLVILDIGGRTWNICVSSGGKC
pCPPB-1_0        CFNFNLFEVNKNIKINKVATVAEGLSSFYTLPKEERAKDLVILDIGGRTWNICVSSGGKC
pCPPB-1_63       CFNFNLFEVNKNIKINKVATVAEGLSSFYTLPKEERAKDLVILDIGGRTWNICVSSGGKC
pJIR3535_00004 SFNFNLFEVTKNIKINKVATVAEGLSSFYTLPKEERAKDLVILDIGGRTWNICVSSGGKC
pNetB-NE10_5     SFNFNLFEVTKNIKINKVATVAEGLSSFYTLPKEERAKDLVILDIGGRTWNICVSSGGKC
pCPF5603_16      SFNFNLFEVTKNIKINKVATVAEGLSSFYTLPKEERVKDLVILDIGGRTWNICVSSGGKC
                 .***.****.**************************.***********************

pCPF4969_61      IMKFTVPGGMIDLYSEIQEDYNKLGNNADVEEIIRLIKNDTIDASKAKERFVKDKLNKVR
pCPPB-1_0        IMKFTVPGGMIDLYSEIQEDYNKLGNNADVEEIIRLIKNDTIDASKAKERFVKDKLNKVR
pCPPB-1_63       IMKFTVPGGMIDLYSEIQEDYNKLGNNADVEEIIRLIKNDTIDASKAKERFVKDKLNKVR
pJIR3535_00004 IMKFTVPGGMIDLYSEIQEDYNKLGNNADVEEIIRLIKNDTIDTSKAKERFVKDKLNKVR
pNetB-NE10_5     IMKFTVPGGMIDLYSEIQEDYNKLGNNADVEEIIRLIKNDTIDTSKAKERFVKDKLNKVR
pCPF5603_16      IMKFTVPGGMIDLYSEIQEDYNKLGNNADVEEIIRLIKNDTIDASKAKERFVKDKLNKVR
                 *******************************************:****************

pCPF4969_61      LKVPNFSTYKIWGAGGGSLDLQDALRENLGNLNFVPDPLFSNVKGNKLIAEAKWGK----
pCPPB-1_0        LKVPNFSTYKIWGAGGGSLDLQDALRENLGNINFVPDPLFSNVKGNKLIAEAKWGKLWQI
pCPPB-1_63       LKVPNFSTYKIWGAGGGSLDLQDALRENLGNINFVPDPLFSNVKGNKLIAEAKWGK----
pJIR3535_00004 LKVPNFSTYKIWGAGGGSLDLQDALRENLGNINFVPDPLFSNVKGNKLIAEAKWGK----
pNetB-NE10_5     LKVPNFSTYKIWGAGGGSLDLQDALRENLGNINFVPDPLFSNVKGNKLIAEAKWGK----
pCPF5603_16      LKVPNFSTYKIWGAGGGSLDLQDALRENLGNINFVPDPLFSNVKGNKLIAEAKWGK----
                 *******************************:************************

**Rep (pNetB-NE10_orf6)**

pCPPB-1_62        MKGVIFMAYIHLLNKEQLEQEKRFNLEERDFHVLNQIEYKESRRKAQSILRFIRKGILLN
pCPF5603_15       ------MAYTHLLTKEQLEQEKRFNLEERDFHVLNQIEYKESRRKAQSILRFIRKGILLN
pJIR3535_00005  ------MAHIHLLTKEQLEQEKRFNLEERDFHVLNQIEYKESRRKAQSILRFIRKGILLN
pCW3_0014         MKGVIFMAHIHLLTKEQLEQEKRFNLEERDFHVLNQIEYKESRRKAQSILRFIRKGILLN
pJIR3844_00007    ------MAHIHLLTKEQLEQEKRFNLEERDFHVLNQIEYKESRRKAQSILRFIRKGILLN
pCpb2-CP1_6       ------MAHIHLLTKEQLEQEKRFNLEERDFHVLNQIEYKESRRKAQSILRFIRKGILLN
p8533etx_52       ------MARIHLLTKEQLEQEKRFNLEERDFHVLNQIEYKESRRKAQSILRFIRKGILLN
pNetB-NE10_6      ------MAHIHLLTKEQLEQEKRFNLEERDFHVLNQIEYKESRRKAQSILRFIRKGILLN

pCPF4969_01 ------MAHIHLLTKEQLEQEKRFNLEERDFHVLNQIEYKESRRKAQSILRFIRKGILLN
                        **  ***.**********************************************

pCPPB-1_62        NGSWSISFSKIHKDYNDWVNKKKRKRPELRNISLKQIKNIVNKLKDLGLLIIENVKKRNC
pCPF5603_15       NGSWSISFSKIHKDYNDWVNKNKKKRPELRNISLKQIKNIVNKLKDLGLLIIENVKKRNC
pJIR3535_00005  NGSWSISFSKIHKDYNDWVNKKKKKRPELKNISLKQIKNIVNKLKDLGLLIIENVKKRNC
pCW3_0014         NGSWSISFSKIHKDYNDWVNKKKKKRPELKNISLKQIKNIVNKLKDLGLLIIENVKKRNC
pJIR3844_00007    NGSWSISFSKIHKDYNDWVNKKKKKRPELKNISLKQIKNIVNKLKDLGLLIIENVKKRNC
pCpb2-CP1_6       NGSWSISFSKIHKDYNDWVNKKKKKRPELKNISLKQIKNIVNKLKDLGLLIIENVKKRNC
pCP8533etx_52     NGSWSISFSKIHKDYNDWVNKKKKKRPELKNISLKQIKNIVNKLKDLGLLIIENVKKRNC
pNetB-NE10_6      NGSWSISFSKIHKDYNDWVNKKKKKRPELKNISLKQIKNIVNKLKDLGLLIIENVKKRNC

pCPF4969_01 NGSWSISFSKIHKDYNDWVNKKKKKRPELKNISLKQIKNIVNKLKDLGLLIIENVKKRNC
                  *********************:*.*****.******************************

pCPPB-1_62        YFLPLPNKLPNNENITMPDTTSIEGNQTTPRYIRNNNIDIDSNSNSKEFNADIYEKCTSL
pCPF5603_15       YFLPLPNKLPNNENITIPDTTSIDANQATPRYIRNNNIDIDSNSNSKEFNADMYEKCTSL
pJIR3535_00005  YFLPLPNKLPNNENITIPDTTSIEGNQTTPRYIRNNNIDIDSNSNSKEFNADMYEKCTSL
pCW3_0014         YFLPLPNKLPNNENITIPDTTSIEGNQATPRYIRNNNIDIDSNSNSKEFNADMYEKCTSL
pJIR3844_00007    YFLPLPNKLPNNENITTPDTTSIEGNQATPRYIRNNNIDIDSNSNSKEFNADMYEKCTSL
pCpb2-CP1_6       YFLPLPNKLPNNENITTPDTTSIEGNQATPRYIRNNNIDIDSNSNSKEFNADMYEKCTSL
pCP8533etx_52     YFLPLPNKLPNNENITMPDTTSIEGNQATPRYIRNNNIDIDSNSNSKEFNADMYEKCTSL
pNetB-NE10_6      YFLPLPNKLPNNENITTPDTTSIEGNQATPRYIRNNNIDIDSNSNSKEFNADMYEKCTSL

pCPF4969_01 YFLPLPNKLPNNENITTPDTTSIEGNQTTPRYIRNNNIDIDSNSNSKEFNADMYEKCTSL
                  **************** ******:.**:************************:*******

pCPPB-1_62        VDVRNKVKELLKAARVKSSWIKNKVLTKLSENYRNITVKFLESYINTVIEDTRNKYYSNY
pCPF5603_15       VDVRNKVKELLKAARVKSSWIKNKVLTKLSENYRNITVKFLESYINTVIENTRNTYYSNY
pJIR3535_00005  VDVRSKVKELLKAARVKSSWIKNKVLTKLSENYRNITVKFLESYINTVIENTRNTYYSNY
pCW3_0014         VDVRNKVKELLKAARVKSSWIKNKVLTKLSENYRNITVKFLESYINTVIENTRNTYYSNY
pJIR3844_00007    VDVRNKVKELLKAARVKSSWIKNKVLTKLSENYRNITVKFLDSYINTVIENTRNTYYSNY
pCpb2-CP1_6       VDVRNKVKELLKAARVKSSWIKNKVLTKLSENYRNITVKFLDSYINTVIENTRNTYYSNY
pCP8533etx_52     VDVRNKVKELLKAARVKSSWIKNKVLTKLSENYRNITVKFLESYINTVIENTRNTYYSNY
pNetB-NE10_6      VDVRNKVKELLKAARVKSSWIKNKVLTKLSENYRNITVKFLESYINTVIENTRNTYYSNY

pCPF4969_01 VDVRNKVKELLKAARVKSSWIKNKVLTKLSENYRNITVKFLESYINTVIENTRNTYYSNY
                  ****.************************************:********:***.*****

pCPPB-1_62        RKYI-NNANDRVLPNFTERNYSNDYWKYLEENLCFN
pCPF5603_15       RKYIKNNANNRALPNFTERNYSKDYWKYLEENLCFN
pJIR3535_00005  RKYIKNNANNRVLPNFTERNYSKDYWKYLEENLCFN
pCW3_0014         RKYIKNNANNRALPNFTERNYSKDYWKYLEENLCFN
pJIR3844_00007    RKYINNNANNRVLPNFTERNYSKDYWKYLEENLCFN
pCpb2-CP1_6       RKYINNNANNRVLPNFTERNYSKDYWKYLEENLCFN
pCP8533etx_52     RKYIKNNANNRVLPNFTERNYSKDYWKYLEENLCFN
pNetB-NE10_6      RKYIKNNANNRVLPNFTERNYSKDYWKYLEENLCFN

pCPF4969_01 RKYIKNNANNRVLPNFTERNYSKDYWKYLEENLYFN
                  **** ****:*.**********:**********.**

Top of Form

Bottom of Form

**RegCB (pNetB-NE10_orf7)**

pCPF5603_14       -MIEIELHNLALNIKEFRKKNKLSQAELAEKLGVARTTIGYYERAEVEPNIYTLIQLSKL
pCW3_0015         -MIEIELHNLALNIKEFRKKNKLSQAELAEKLGVARTTIGYYERAEVEPNIYTLVQLSKL
pCPPB-1_61        MAKEIGLHRLATTLKDFRKDNKISQEDFAKQLEIARSTLSYYERAKSEPPIYTLVKMSEV
pCPF4969_1        MAKEIGLHKLATTLKDFRKDNKISQEDFAKQLEIARSTLSYYERAKSEPPIYTLVKMSEV
p8533etx_53       MAKEIGLHRLATTLKDFRKDNKISQEDFAKQLEIARSTLSYYERAKSEPPIYTLVKMSEV
pJIR3535_00006  MTKEIGLHRLATTLKDFRKDNKISQEDFAKQLEIARSTLSYYERAKSEPPIYTLVKMSEV
pNetB-NE10_7      MTKEIGLHRLATTLKDFRKDNKISQEDFAKQLEIARSTLSYYERAKSEPPIYTLVKMSEV
                    ** **.** .:*:***.**:** ::*::* :**:*:.*****: ** ****:::*::

pCPF5603_14      MNRSIDSLLGLNHSTENTND--LNNSDLSKKIFILNKLIEKNNQSF--------------
pCW3_0015        MNRSIDSLLGLNHPNETTND--LNNSDLSKKIFILNKLIEKNTQSF--------------
pCPPB-1_61       MNCSIDELLGTTKEIASSTKEKFSYSELTEKIYYLNELIDKNIKTYEDLIMSKKRTERML
pCPF4969_1       MNCSIDELLGTTKAISNSAEEKFSCNELIEKIYYLNELIDKNRKNYEDLIMSKKRTERMF
pCP8533etx_53    MNCSIDELLGTTKAISNSAEEKFSYNELIEKIYYLNELIDKNRKNYEDLIMSKKRTERML
pJIR3535_00006 MNCSIDELLGTTKAITNSDKEKFSYNELTEKIYYLNELIDRNRKNYEDLIMSKKRTERMI
pNetB-NE10_7     MNCSIDELLGTTKAITNSDEEKFSYNELTEKIYYLNELIDRNRKNYEDLIMSKKRTERMI
                 ** ***.*** .:   .: .  :. .:* :**: **:**:.* :.:              

pCPF5603_14      --------------KELESSKLRTERMF----------------------------NELK
pCW3_0015        --------------NDLETSKLRTERMFNELQMSKKRTERMF--------------NELK
pCPPB-1_61       --------------DELSMSKKRTERMFDELLMSKKRTERMF--------------TELT
pCPF4969_1       DELSMSKKRTERMFDELSMSKKRTERMFDELSMSKKRTERMFDELSMSKKRTERMFIELT
pCP8533etx_53    DELSMSKKRTERMFDELSMSKKRTERMFDELLMSKKRTERMF--------------IELN
pJIR3535_00006 --------------DELSMSKKRTERMFDELLMSKKRTERMF--------------IELN
pNetB-NE10_7     --------------DELSMSKKRTERMFDELLMSKKRTERMF--------------IELN
                               .:*. ** ******                             **.

pCPF5603_14      RTRNRAENDIKLFQELSKEFTNTLDKEHEINLSDALEYQDIN----YNFSTTEKEDFLSS
pCW3_0015        RTRNRAENDIKLFQELSKEFTNTLDKEHETDLSDALEYQDIN----YNFSTTEKEDFLSS
pCPPB-1_61       RVLNRSESDIKTFQKLSKEFASLLDKNKSIENEYSKSIKEVNEDNIFNYSITEKEDFLSS
pCPF4969_1       RVLNRSESDIKTFQKLSKEFATLLDKNKSIENEYSKSIKEVNEDNIFNYSITEKEDFLSS
pCP8533etx_53    RITNRSESDILTFQKLSKEFASLLDKNKSIENEYSKSIKEVNEDNIFNYSITEKENFLSS
pJIR3535_00006 RITNRSESDILTFQKLSKEFASLLDKNKSIENEYSKSIKEVNEDNIFNYSITEKENFLSS
pNetB-NE10_7     RITNRSESDILTFQKLSKEFASLLDKNKSIENEYSKSIKEVNEDNIFNYSITEKENFLSS
                 *  **:*.**  **:*****:. ***::. : . : . :::*    :*:* ****:****

pCPF5603_14      NLKDLISNLNFIPIDVVGEVSCGTPSYAFNEISKSIALPSNYKDCFALRVKGNSMNKLFK
pCW3_0015        NLKDLISNLNFIPIDVVGEVSCGTPSYAFNEISKSIALPSNYKDCFALRVKGNSMNKLFK
pCPPB-1_61       NLKNLISNLNFIPIDVVGEVSCGTPSYAFNEISKSIALPSNYKDCFALRVKGNSMNKLFK
pCPF4969_1       NLKDLISNLNFIPIDVVGEVSCGTPSYAFNEISKSIALPSNYKDCFALRVKGNSMNKLFK
pCP8533etx_53    NLKDLISNLNFIPIDVVGEVSCGTPSYAFNEISKSIALPSNYKDCFALRVKGNSMNKLFK
pJIR3535_00006 NLKDLISNLNFIPIDVVGEVSCGTPSYAFNEISKSIALPSNYKDCFALRVKGNSMNKLFK
pNetB-NE10_7     NLKDLISNLNFIPIDVVGEVSCGTPSYAFNEISKSIALPSNYKDCFALRVKGNSMNKLFK
                 ***:********************************************************

pCPF5603_14      DNELIICCRNKTPIDGDIVIAYLSENNEATCKKIHKKKDKLELHPCSTLPYEIQYYDKNS
pCW3_0015        DNELIICCRNKTPIDGDIVIAYLSENNEATCKKIHKKKDKLELHPCSTLPYEIQYYDKNS
pCPPB-1_61       DNELIICCRNKTPIDRDIVIAYLSENNEATCKKIHKKKDKLELHPCSTLPYEIQYYDKNS
pCPF4969_1       DNELIICCRNKTPIDGDIVIDYLSENNEATCKKIHKKKDKLELHPCSTLPYEIQYYDKNS
pCP8533etx_53    DNELIICCRNKTPIDGDIVIASLSENNEATCKKIHKKKDKLELHPCSTLPYEIQYYDKNS
pJIR3535_00006 DNELIICCRNKTPIDGDIVIAYLSENNEATCKKIHKKKDKLELHPCSTLPYEIQYYDKNS
pNetB-NE10_7     DNELIICCRNKTPIDGDIVIAYLSENNEATCKKIHKKKDKLELHPCSTLPYEIQYYDKNS
                 *************** ****  **************************************

pCPF5603_14      DINIIGVVLGSLTDILDLENIDIEYLEEKLNTI
pCW3_0015        DINIIGVVLGSLNDILDLENIDIEDLEEKLNTI
pCPPB-1_61       DINIIGVVLGSLNDILDLENIDIEDLEEKLNTI
pCPF4969_1       DINIIGVVLGSLNDILDLENIDIEDLKEKLNTI
pCP8533etx_53    DINIIGVVLGSLTDILDLENIDIEDLEEKLNTI
pJIR3535_00006 DINIIGVVLGSLTDILDLENIDIEDLEEKLNTI
pNetB-NE10_7     DINIIGVVLGSLTDILDLENIDIEDLEEKLNTI
                 ************.*********** *:******

**RegD (pNetB-NE10_orf8)**

pCPF469_2         MKIWIKVINMENKLSFLRKNKVLSQREVASLIGKEARFIHKLENGKVKNPSASEVYKLSR
pCPPB-1_60        MKIWIKVINMENKLSFLRKNKVLSQREVASLIGKEARFIHKLENGKVKNPSASEVYKLSR
p8533etx_54       MKIWIKVINMENKLSFLRKNKVLSQREVASLIGKEARFIHKLENGKVKNPSASEVYKLSR
pCPF5603_13       MKIWIKMINMENKLSFLRKNKVLSQREVASLIGKEARFIHKLENGKVKNPSASEVYKLSR
pCW3_0016         MKIWIKVINMENKLSFLRKNKVLSQREVASLIGKEARFIHRLENGKVKNPTASEVYKLSR
pJIR3535_00007  ---------MENKLSFLRKNKVLSQREVASLIGKEARFIHRLENGKVKNPTASEVYKLSR
pNetB-NE10_8      ---------MENKLSFLRKNKVLSQREVASLIGKEARFIHRLENGKVKNPTASEVYKLSR
pCpb2-CP1_8       ---------VENKLSFLRKNKVLSQREVASLIGKEARFIHKLENGKVKNPTASEVYKLSR
pJIR3844_00009    ---------MENKLSFLRKNKVLSQREVASLIGKEARFIHKLENGKVKNPTASEVYKLSR
                           :******************************.*********:*********

pCPF4969_2        IYDTSMEEIYRAIVLNN
pCPPB-1_60        IYDTSMEEIYRAIVLNN
pCP8533etx_54     IYDTSMEEIYRAIVLNN
pCPF5603_13       IYDTSMEEIYRAIVLNN
pCW3_0016         IYDTSMEEIYRAIVLNN
pJIR3535_00007  IYDTSMEEIYRAIVLNN
pNetB-NE10_8      IYDTSMEEIYRAIVLNN
pCpb2-CP1_8       IYDTSMEEIYRAIVLNN
pJIR3844_00009    IYDTSMEEIYRAIVLNN
                  *****************
