# Supplementary material for: Sequence of Two Plasmids from Clostridium perfringens Chicken Necrotic Enteritis Isolates and Comparison with C. perfringens Conjugative Plasmids
Source: PLoS One. 2012 Nov 26;7(11):e49753. doi: 10.1371/journal.pone.0049753 (PMC3506638; doi:10.1371/journal.pone.0049753)
Supplement: Figure S3 — Repeats found on the upstream region of parM gene. Possible tandem repeats found on the upstream region of parM gene next to rep gene from C. perfringens plasmids using etandem (http://emboss.bioinformatics.nl/cgi-bin/emboss/etandem). (DOCX) [file pone.0049753.s003.docx]

**Figure S3. Possible tandem repeats found on the upstream region of *parM* gene next to *rep* gene from *C. perfringens* plasmids using etandem (**[**http://emboss.bioinformatics.nl/cgi-bin/emboss/etandem**](http://emboss.bioinformatics.nl/cgi-bin/emboss/etandem)**).**

**pNetB-NE10**

***parM* gene:2219-3064**

***rep* gene:3569-4381**

Start End Strand Score Size Count Identity Consensus

3162 3227 + 23 11 6 75.8 tactttaaagt

3097 3151 + 22 11 5 80.0 aagtaacttaa

**pJIR3535**

***parM* gene:2246-3091**

***rep* gene: 3597-4409**

Start End Strand Score Size Count Identity Consensus

3124 3178 + 22 11 5 80.0 aagtaacttaa

3189 3254 + 22 11 6 75.8 tactttaaagt

**pCpb2-CP1**

***parM* gene:2537-3454**

***rep* gene: 4050-4867**

Start End Strand Score Size Count Identity Consensus

3613 3727 + 20 23 5 68.7 atattcagtatatataaatgtat

**pJIR3844**

***parM* gene: 2545-3462**

***rep* gene: 4058-4870**

Start End Strand Score Size Count Identity Consensus

3611 3714 + 21 13 8 66.3 tatatatattata

**pCPF4969**

***parM* gene:68546-69406**

***rep* gene: 69897-70480**

Start End Strand Score Size Count Identity Consensus

69419 69473 + 20 11 5 78.2 ttataagtgac

**pCPF5603**

***rep gene* : 12651-13463**

***parM gene*: 13972-14817**

Start End Strand Score Size Count Identity Consensus

13884 13938 + 22 11 5 80.0 tttaagtcact

**pCPPB-1**

***rep* gene: 55506-56333**

***parM* gene:56830-57675**

Start End Strand Score Size Count Identity Consensus

56742 56807 + 29 11 6 80.3 acttataagtt

56662 56716 + 20 11 5 78.2 taaaagtgact

**pCP8533etx**

***parM* gene: 53274-54152**

***rep* gene: 54716-55528**

Start End Strand Score Size Count Identity Consensus

54242 54301 + 26 10 6 80.0 attaattaat

DNA sequence alignment of upstream region of *parM* gene (MUSCLE-3.7)

pNetB_5          --------------ACTTTTTTCAATAGCTTTGATATCATAAAGCTATGAAAAGTGAATA

pJIR3535_00004 ---------------CTTTTTTCAATAGCTTTGATATTATAAAGCTATGAAAAGTGAATA
pCPF5603_16      ------------------TTTTCAATAGCTTTGATATAGTAAAGCTATGAAAAGTGAATA
pCPF4969_61      AATAGATATCATTGACTTTTTTCAATAGCTTTGATATCATAAAGCTATGAAAAGTGAATA
pCPPB-1_63       ------------------------ATAGCTTTGATATTATAAAGCTATGAAAAGTGAATA
                                         *************  *********************

pNetB_5          TTTCAAATAGAACTTTGTTCTATTTCGGATTTAAGTTCATCCATTAAGTTTACTTCGATT

pJIR3535_00004 TTTCAAATAGAACAAAGTTCTATTTAGGATTTAGGTTCATCCATTAAGTTTACTTCGATT
pCPF5603_16      TTTCAAATAGAACTTTGTTCTATTTCGGATTTAGGTTCATCCATTAAGTTTACTTACTTT
pCPF4969_61      TTTCAAATAGAACTTTGTTCTATTTAGGATTTAGGTTCATCCATTAAGTTTACTTTGTTT
pCPPB-1_63       TTTCAAATAGAACTTTGTTCTATTTAGGATTTAGGTTCATCCATTAAGTTTACTTTGTTT
                 ************************* ******* *********************   **

pNetB_5          GCCGTCT-GGGGAAACTTAATGGATTTTTCCTTTTCCGTACTATTTAATTTAATTGAGTT

pJIR3535_00004 GCCGTCT-GGGGAAACTTAATGGATTTTTCCTTTTCCGTACTATTTAATTTAATTGAGTT
pCPF5603_16      GGCGAGTCGAGGAAACTTAGTGGATTTTTCCTTTTCCGTACTATTTAATTTAATTGAGTT
pCPF4969_61      GGCGACT--AGGAAACTTAATGGATTTTTCCTTTTTCGTACTATTTAATTTAATTGAGTT
pCPPB-1_63       GGCGACT--AGGAAACTTAATGGATTTTTCCTTTTCCGTATTATTTAATTTAATTGAGTT
                 * **  *   ********* *************** **** *******************

pNetB_5          AATAATACCATGTTAAAAATAATTTTTCAAGAGAGTAAAATTATAGAAAATTTTACTCTC

pJIR3535_00004 AATAATACCATGTTAAAAATAATTTTTCAAGAGAGTAAAATTATAGAAAATTTTACTCTC
pCPF5603_16      AATAATACCATGTTAAAAATAATTTTTCAAGAGAGTAAAATTATAGAAAATTTTACTCTC
pCPF4969_61      AATAATACCATGTTAAAAATAATTTTTCAAGAGAGTAAAATTATAGAAAATTTTACTCTC
pCPPB-1_63       AATAATACCATGTTAAAAATAATTTTTCAAGAGAGTAAAATTATAGAAAATTTTACTCTC
                 ************************************************************

pNetB_5          TTTTTTATTA-ATTTTTT--AAAGTTACTTAAAAGTAACTTTAAAGTAATTAAAAGTTGA

pJIR3535_00004 TTTTTTATTATATTTTTT--AAAGTTACTTAAAAGTAACTTTAAAGTAATTAAAAGTTGA
pCPF5603_16      TTTTTTATTATATTTTTTCTAAAGTCACTTAAAAGTAACTTTTTGGTGATTAAAAGTTGA
pCPF4969_61      TTTTTCATTACATTTTTCTAAAAGTAACTTAAAAGTAACTTTTAGGTGGTTAAAAGTTGA
pCPPB-1_63       TTTTTTATTATATTTTTCTAAAAGTCACTTAAAAGTAACTTTTTGGTGATTAAAAGTTGA
                 ***** ****  *****   ***** ****************   **  ***********

pNetB_5          CTTTTAGGTAACTTTTTGATATTATAATGATATAAGTTACTTAAAAATAACTTTTTAAAC

pJIR3535_00004 CTTTTAGGTAACTTTTTGATATTATAATGATATAAGTTACTTAAAAATAACTTTTTAAAC
pCPF5603_16      CTTTTAGGTAACTTTTTGATATTATAATAATATAAGTCACTTAAAAGTAACTTTTTAAAC
pCPF4969_61      CTTTTAAGTTACTTTATGATATTATAATAGTATAAGTCACTTAAAAGTCACTTATAAAAC
pCPPB-1_63       CTTTTAAGTGACTTTTTGTTATAATAATAGCATTAGTCACTTAAAGGTAACTTATAAAAT
                 ****** ** ***** ** *** *****   ** *** *******  * **** * *** 

pNetB_5          ACTTTAAAGTTACTTTTAAGTAACTTATGATTT--------TTTTGAAAAAATGGAGGTT

pJIR3535_00004   ACTTTAAAGTTACTTTTAAGTAACTTATGATTT--------TTTTGAAAAAATGGAGGTT
pCPF5603_16      ACTTTAAAATTACTTTTAAGTGACTTATGATTT--------GTTTTAAAAAATGGAGGTT
pCPF4969_61      ACTTTAAAGTAACTTTTGAGTAACCGATGATTT---------TTTAAAAAA---------
pCPPB-1_63       ACTTTAAAGTTACTTTAAAGTTACTTTTAAGTTGCTAATAATTTTAAAAAAATGGAGGTT
                 ******** * *****  *** **   * * **         *** *****         

pNetB_5          TTAAAA

pJIR3535_00004 TTAAAA
pCPF5603_16      TTAAAA
pCPF4969_61      ------
pCPPB-1_63       TTAAAA

pJIR3844_00006    AAGTTTTCCCAACTCGCCAAAGTAAGTAAACTTAATGGATTTTTCCTTTTCCGTACTATT
pCpb2_5           AAGTTTTCCCAACTCGCCAAATTAAGTAAACTTAATGGATTTTTCCTTTTCCGTACTATT
                  ********************* **************************************

pJIR3844_00006    TAATTTAATTGAGTTAATAATACCATGTTAAAAATAATTTTTCAAGAGAGTAAAATTATA
pCpb2_5           TAATTTAATTGAGTTAATAATACCATGTTAAAAATAATTTTTCAAGAGAGTAAAATTATA
                  ************************************************************

pJIR3844_00006    GAAAATTTTACTCTCTTTTTTTATTAATTTCAATATATGTATATACTGAATATATACATA
pCpb2_5           GAAAATTTTACTCTCTTTTTTTATTAATTTCAATATATGTATATACTGAATATATACATA
                  ************************************************************

pJIR3844_00006    TATTGAAATTAATAAAAAGCAATTATATATACTGAGTATATATAATTATATTTTATATAT
pCpb2_5           TATTGAAATTAATAAAAAGCAATTATATATACTGAGTATATATAATTATATTTTGTATAT
                  ****************************************************** *****

pJIR3844_00006      ATTCAGTATATACAAAAGTGAGAAAATTAAAGAGAATAATAGTATTCTGAAGAAAAATCA
pCpb2_5           ATTCAGTATATACAAAAGTGAGAAAATTAAAGAGAATAATAGTATTCTGAAGAAAAATCA
                  ************************************************************

pJIR3844_00006    GTATAAACTCAGTATATACATGGTTGAAAAGTTGTTTTTAGTATATATCTAGTATATACT
pCpb2_5           GTATAAACTCAGTATATACATGGTTGAAAAGTTGTTTTTAGTATATATCTAGTATATACT
                  ************************************************************

pJIR3844_00006    AAATTTATAAAAGATAATTAATTTTGAAAGGAGCATTAAA
pCpb2_5           AAATTTATAAAAGATAATTAATTTTGAAAGGAGCATTAAA
                  ****************************************

pCP8533etx_52    ACAATACCGACATTAAATAATATTATT-------------------TGATATTATTTAAT
pCW3_0014        --ATTTTC--CGTAAAATTGTGATGTTTTTTTCATTTTCAACAAACTGTCATTACGTAAA
                   * *  *  * * ****  *  * **                   **  ****  *** 

pCP8533etx_52    ATTATTCGATTTTCA----AAACAAAATAAAAAGAGAGTAAAATTTTCTATAATTTTACT
pCW3_0014        ATTGTGATGTTTTTAATTGAGGCTAAAATAAAAGAGAGTAAAATTTTCTATAATTTTACT
                 *** *    **** *    *  * ***  *******************************

pCP8533etx_52    CTCTTGAAAAATTATTTTTAACATGGTATTATTAACTCAATTAAATTAAATAGTACGGAA
pCW3_0014        CTCTTGAAAAATTATTTTTAACATGGTATTATTAACTCAATTAAATTAAATAGTACGGAA
                 ************************************************************

pCP8533etx_52    AAGGAAAAATCCATTAAGTTTTCCCAACTCGCCAAAGTAAGTAAACTTAATGGATGAACT
pCW3_0014        AAGGAAAAATCCATTAAGTTTTCTAGAA-CGCCAATCAAAGTAAACTTAATGGATGAACT
                 ***********************   *  ******   **********************

pCP8533etx_52    TAAATCCGAAATAGAACAAAGTTCTATTTGAAATATTCACTTTTCATAGCTTTATGATAT
pCW3_0014        TAAATCCGAAATAGAACAAAGTTCTATTTGAAATATTCACTTTTCATAGCTTTATGATAT
                 ************************************************************

pCP8533etx_52    CAAAGCTATTGAAAAAAGTCAATGATATCTATTGACTTTTTAATAGAAATGTAATATTAG
pCW3_0014        CAAAGCTATTGAAAAAAGTCAATGATATCTATTGACTTTTTAATAGAAATGTAATATTAG
                 ************************************************************

pCP8533etx_52    CAAATTTTAACTTCTTTCTATTTAGGATTTAGTGTATTTTCCTAGATGAAAGGAGTTATT
pCW3_0014        CAAATTTTAACTTCTTTCTATTTAGGATTTAGTGTAATTTCCTAG---------------
                 ************************************ ********               

pCP8533etx_52    TTT
pCW3_0014        ---
