# Supplementary material for: Sequence of Two Plasmids from Clostridium perfringens Chicken Necrotic Enteritis Isolates and Comparison with C. perfringens Conjugative Plasmids
Source: PLoS One. 2012 Nov 26;7(11):e49753. doi: 10.1371/journal.pone.0049753 (PMC3506638; doi:10.1371/journal.pone.0049753)

**Figure S4 (A). PFGE analyses of plasmids from healthy *C. perfringens* poultry strains**. Agarose plugs containing DNA from each specified isolate were digested with *Not*I and subjected to PFGE and staining with ethidium bromide. See Table1 and 2 for isolate features. Line numbers indicate isolate numbers M: Mid-Range II PFG molecular DNA ladder (Kb).

***netB+ netB***-


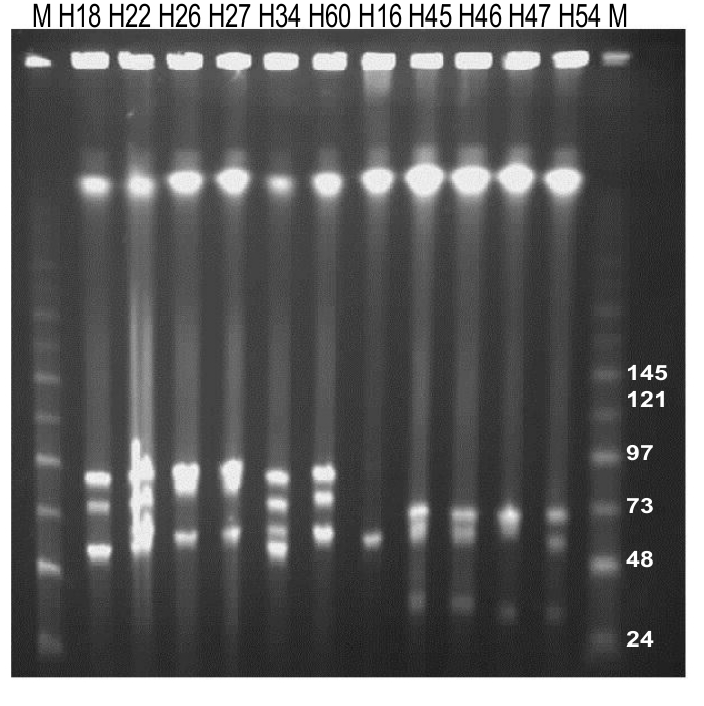


**Figure S4 (B). PFGE Southern blot of plasmids from healthy *C. perfringens* poultry strains**. Southern blotting of PFGE (Figure 4A) was performed with *only* DIG-labelled probe for ***cpb2*** gene. M: Mid-Range II PFG molecular DNA ladder (Kb).


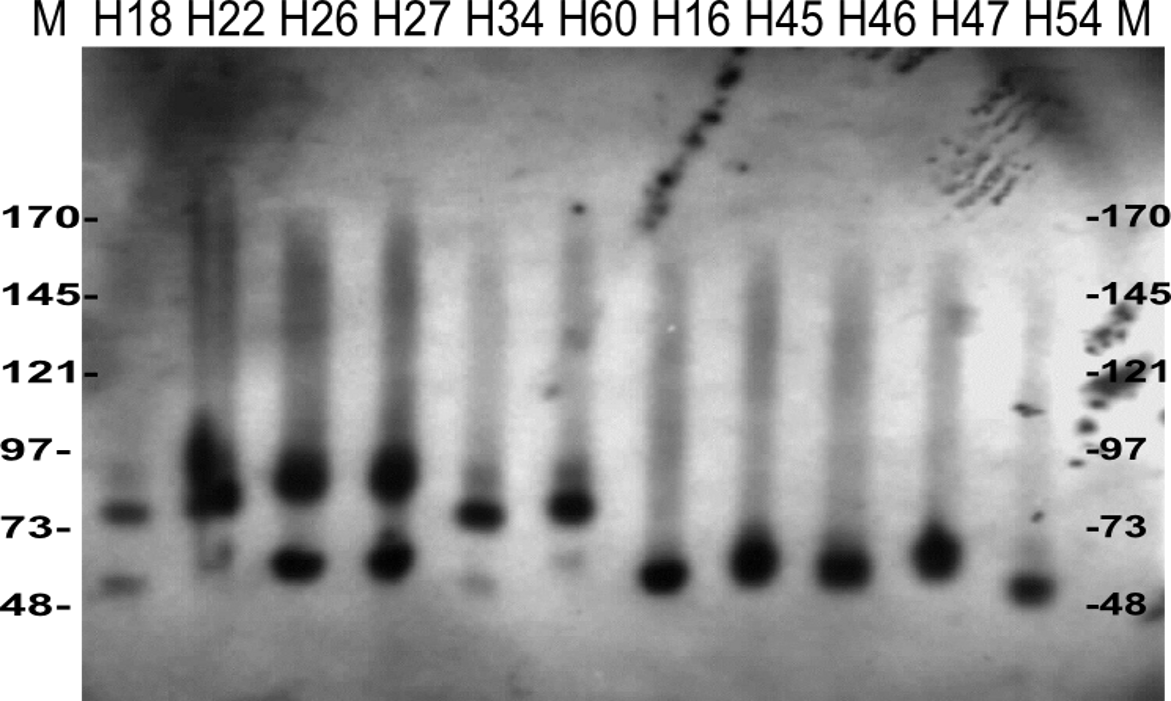


**Figure S4 (C)**. **PFGE Southern blot of plasmids from healthy *C. perfringens* poultry strains**. Southern blotting of PFGE (Figure 4A) was performed with *only* DIG-labelled probe for ***netB*** gene. M: Mid-Range II PFG molecular DNA ladder (Kb).


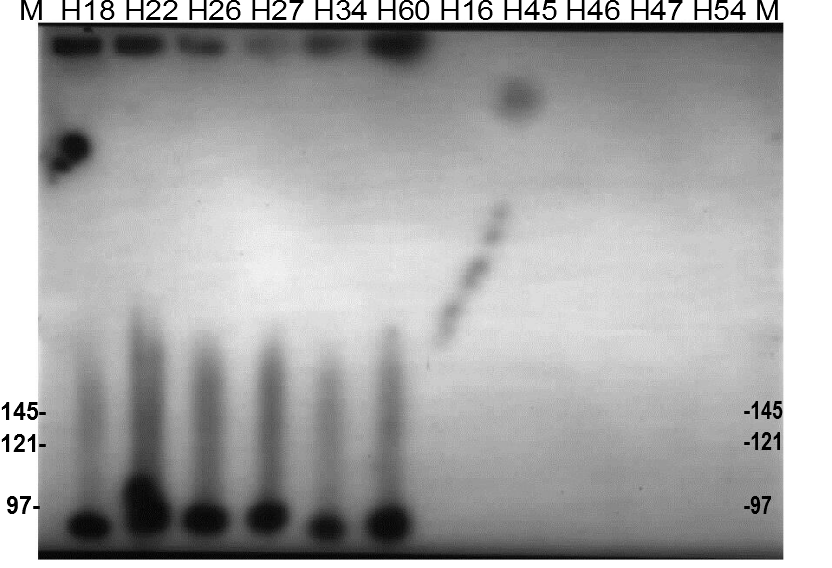

Supplement: Figure S4 — PFGE and Southern blot analyses of plasmids from healthy C. perfringens poultry strains. (A) PFGE analyses of plasmids from healthy C. perfringens poultry strains. Agarose plugs containing DNA from each specified isolate were digested with NotI and subjected to PFGE and staining with ethidium bromide. See Table1 and 2 for isolate features. Line numbers indicate isolate numbers M: Mid-Range II PFG molecular DNA ladder (Kb). (B) PFGE Southern blot of plasmids from healthy C. perfringens poultry strains. Southern blotting of PFGE (Figure S4A) was performed with only DIG-labelled probe for cpb2 gene. M: Mid-Range II PFG molecular DNA ladder (Kb). (C) PFGE Southern blot of plasmids from healthy C. perfringens poultry strains. Southern blotting of PFGE (FigureS 4A) was performed with only DIG-labelled probe for netB gene. M: Mid-Range II PFG molecular DNA ladder (Kb). (DOCX) [file pone.0049753.s004.docx]
