# Supplementary material for: Sequence of Two Plasmids from Clostridium perfringens Chicken Necrotic Enteritis Isolates and Comparison with C. perfringens Conjugative Plasmids
Source: PLoS One. 2012 Nov 26;7(11):e49753. doi: 10.1371/journal.pone.0049753 (PMC3506638; doi:10.1371/journal.pone.0049753)

**Figure S5**. **PFGE analyses of plasmids from *C. perfringens* strains**. Agarose plugs containing DNA from each specified isolate were digested with *Not*I and subjected to PFGE and staining with ethidium bromide. Lines indicate: CW504 recipient strain (plasmid free); T98 (transconjugant carrying the plasmid pCpb2); T117 (transconjugant carrying three CP1 plasmids) ; T118 (transconjugant carrying four CP1 plasmids) ; T119 (transconjugant carrying two CP1 plasmids); T125(transconjugant carrying two CP1 plasmids) ; T128 (transconjugant carrying two CP1 plasmids); CP1 donor strain (harbours four large plasmids); M: Mid-Range II PFG molecular DNA ladder (Kb).


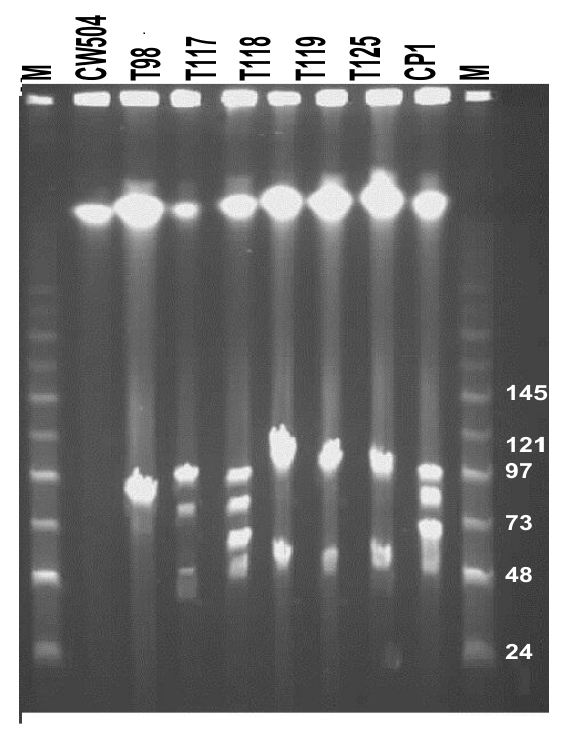

Supplement: Figure S5 — PFGE analyses of plasmids from transconjugants C. perfringens strains. Agarose plugs containing DNA from each specified isolate were digested with NotI and subjected to PFGE and staining with ethidium bromide. Lines indicate: CW504 recipient strain (plasmid free); T98 (transconjugant carrying the plasmid pCpb2); T117 (transconjugant carrying three of CP1 plasmids); T118 (transconjugant carrying four of CP1 plasmids); T119 (transconjugant carrying two of CP1 plasmids); T125(transconjugant carrying two of CP1 plasmids); T128 (transconjugant carrying two of CP1 plasmids); CP1 donor strain (harbours four large plasmids); M: Mid-Range II PFG molecular DNA ladder (Kb). (DOCX) [file pone.0049753.s005.docx]
