# Supplementary material for: Sequence of Two Plasmids from Clostridium perfringens Chicken Necrotic Enteritis Isolates and Comparison with C. perfringens Conjugative Plasmids
Source: PLoS One. 2012 Nov 26;7(11):e49753. doi: 10.1371/journal.pone.0049753 (PMC3506638; doi:10.1371/journal.pone.0049753)
Supplement: Table S1 — List of primers. (A) Primers used for PCR DIG labelling and mutation (B) Primers used for overlapping PCR reactions of the three Pathogenicity loci characteristic of necrotic enteritis C. perfringens isolates. (DOCX) [file pone.0049753.s006.docx]

**Table S1 (A). Primers used for PCR DIG labelling and sequencing.**

| **PCR Reaction** | Primer | Primer sequence (5’-3’) | Predict size (bp) | Reference |
| --- | --- | --- | --- | --- |
| **DIG probe *netB*** | AKP78  AKP79 | GCTGGTGCTGGAATAAATGC  TCGCCATTGAGTAGTTTCCC | 384 | Keyburn et al., 2008 |
| **DIG probe *cpb2*** | Cpb2-F  Cpb2-R | AGATTTTAAATATGATCCTAACC  CAATACCCTTCACCAAATACTC | 567 | This study |
| **DIG probe *hdhA*** | HDHA-F  HDHA-R | CAATTGTTACTGCAGCTTCAAGAGG  TAATTTTTACCGCCACCTTCATAATA | 387 | Lepp et al.,2010 |
| **Long Range PCR-5’ NEL_1** | SP-F  DCM-R | GTGCAGTTACTATTATAGGACC  TTGAAGCACTAAGGATTATAGA | 8503 | Lepp et al.,2010 |
| **Long Range PCR-3’ NEL_1** | BLAC-F  LEXA-F | AACTACTTAATAGACACAGGAA  TACAGGATCAGTATCATATACC | 2677 | Lepp et al.,2010 |
| **netB mutant** | NetB_IBS | AAAAAAGCTTATAATTATCCTTAGATGTCTCTAATGTGCGCCCAGATAGGGTG | 341 | This study |
|  | NetB_EBS-2 | TGAACGCAAGTTTCTAATTTCGATTACATCTCGATAGAGGAAAGTGTCT |  |  |
|  | NetB_EBS-1d | CAGATTGTACAAATGTGGTGATAACAGATAAGTCTCTAATTCTAACTTACCTTTCTTTGT |  |  |
| **cpb2 mutant** | cpb2_IBS | AAAAAAGCTTATAATTATCCTTATTAATCGTAAGTGTGCGCCCAGATAGGGTG | 341 | This study |
|  | cpb2_EBS-2 | TGAACGCAAGTTTCTAATTTCGGTTATTAATCGATAGAGGAAAGTGTCT |  |  |
|  | cpb2_EBS-1d | CAGATTGTACAAATGTGGTGATAACAGATAAGTCGTAAGTAATAACTTACCTTTCTTTGT |  |  |
|  | EBS- Universal | CGAAATTAGAAACTTGCGTTCAGTAAAC |  | This study |

**Table S1 (B). Primers used for overlapping PCR reactions of the three pathogenicity loci characteristic of necrotic enteritis *C. perfringens* isolates.**

| **PCR reaction** | **Predicted Size (bp)** | **Primers** | **Primer sequences** |
| --- | --- | --- | --- |
| Long Range PCR-5’ | ~9000 | DCM-F | GTGAATCCTACAAGTCTCCAGC |
|  |  | SIGP-F | GTGCAGTTACTATTATAGGACC |
| NEL1-1 | 3722 | SIGPROT-R | CCAATACTGCATGTTACTTCTA |
|  |  | 03749-R | GTTGTTGTGTAATATATCTAGC |
| NEL1-2 | 4482 | 03749-F | GTATACTTCAGAAGGAACGGCT |
|  |  | SORTA-R | TCTGCTTCACTAAGATCATTAT |
| NEL1-3 | 2737 | SORTA-F | TATTGTCAGCTCATACAGGATT |
|  |  | 1281-R1 | CTACTGTATTCCAAGCTGATCT |
| NEL1-4 | 4869 | 1281-F1 | GGCTATACTTGTCGCATCATTA |
|  |  | 1281-R2 | ACTTCATCATCAGTTGCATCTT |
| NEL1-5 | 5530 | 1281-F2 | CAAGTCTTGAAGAAGTTATAGC |
|  |  | WBRA-R | AGCTTCACCACTACCAATTGAT |
| NEL1-6 | 3000 | WBRA-F | GCAGATTCACTTCCTGTAACAA |
|  |  | LKI-R | CAGTTCATATGTATGTGTTGAC |
| NEL1-7 | 3564 | LKII-F | TTATTCCTTCTTCACTTGTTCA |
|  |  | CHIA-R | AAGGAGAAGCAGTAACAGCAGC |
| NEL1-8 | 2224 | CHIA-F | TGTTGGAGGTTGGACAGGAACA |
|  |  | CHIB-R | CTCCAAGCTCCAGCCATATCAT |
| NEL1-9 | 3942 | CHIB-F | CAACAATAGCCGCAACTCCTTC |
|  |  | tn1546-R | GAAGAACATTATACAGTCATAG |
| NEL1-10 | 3118 | tn1546-F | TTGTCTAGAGTAAGAAGCTAAT |
|  |  | AKP78 | GCTGGTGCTGGAATAAATGC |
| NEL1-11 | 2760 | AKP79 | TCGCCATTGAGTAGTTTCCC |
|  |  | INTER-F | CAACGTTAATAGTAGGTTCAAT |
| NEL1-12 | 4087 | INTER-R | TCTCTAATGGACTTATATCTTC |
|  |  | MPROT-R | TCATTATTATATGCTAATCCAA |
| NEL1-13 | 2926 | MPROT-F | ATTGAATGGATGTATTATGCAG |
|  |  | MAR-R | GTTATAATTGGAGTTAGTGTTC |
| Long Range PCR-3’ | 2700 | BLAC-F | AACTACTTAATAGACACAGGAA |
|  |  | LEXA-R | GGATATCAACCTACGGTTAGAG |
| NEL2-Link 5’ | 928 | 457-F | AGTTGCTGTTATGCTAGATAGG |
|  |  | SigF-R | TGTAGTTACTTCTGAATCATGG |
| NEL2-1 | 2392 | SigF-F | TCCTAAGCATAGAGATACAAGA |
|  |  | FtZ-R | TCTGTACTAATTCCATAAGCAC |
| NEL2-2 | 3037 | FtZ-F | ACAACTGTGCTTATGGAATTAG |
|  |  | CotH-R | TTCTCCATTGGTGTCTATAACT |
| NEL2-3 | 4089 | CotH-F | TTGATAGGCTTATGGAAGACAA |
|  |  | Gh-R | GATATGTTGAAGCATTGATACC |
| NEL2-Link 3’ | 1914 | Gh-F | AAGATCCAGAGGCCTATTAGAA |
|  |  | 469-R | TGCTGAAGCTACTAAGAATAGA |
| NEL3-1 | 2.9 | orf53-F1 | CCAACAACTCCCATAAAAGATG |
|  |  | res-R1 | GCAGTTAATTCTCCGTTTTTCC |
| NEL3-2 | 0.94 | 1852_R2 | CATCCAAGGCTCATCACTCC |
|  |  | 1852-F2 | GAAGTGCCATCTAATTTTGAAGAAA |
| NEL3-3 | 2.9 | 1852_F1 | GGAGTGATGAGCCTTGGATG |
|  |  | 1852_R1 | AAGAGCAAATCCTCCAGCAA |
| NEL3-4 | 1.9 | 5603_56-F1 | TCCTACATGGACAATTGCTGA |
|  |  | 4143_3417-F1 | TTGCTGGAGGATTTGCTCTT |
