# Supplementary material for: Sequence of Two Plasmids from Clostridium perfringens Chicken Necrotic Enteritis Isolates and Comparison with C. perfringens Conjugative Plasmids
Source: PLoS One. 2012 Nov 26;7(11):e49753. doi: 10.1371/journal.pone.0049753 (PMC3506638; doi:10.1371/journal.pone.0049753)
Supplement: Table S2 — Comparison of NE C. perfringens plasmids. (A) Comparison of coding sequences pNetB-NE10 and pJIR3535 NE C. perfringens plasmids by means of BLASTn analyses. Open reading frames are labeled according to the annotation of plasmid pNetB-NE10 (B) Comparison of open reading frames pCpb2-CP1 and pJIR3844 NE C. perfringens plasmids by means of BLASTn analyses. Open reading frames are labeled according to the annotation of plasmid pCpb2-CP1. (DOCX) [file pone.0049753.s007.docx]

**Table S2 (A). Comparison of coding sequences pNetB-NE10 and pJIR3535 NE *C. perfringens* plasmids by means of BLASTn analyses.** Open reading frames are labeled according to the annotation of plasmid pNetB-NE10.

| **CDS/ORF** | **% Identity** |
| --- | --- |
| *orf1* | 99 |
| *regB* | 96 |
| *orf3* | 96 |
| *orf4* | 100 |
| *orf5* | 100 |
| *rep* | 99 |
| *regCB* | 99 |
| *regD* | 100 |
| *orf9* | 100 |
| *orf10* | 99 |
| *orf11* | 100 |
| *cna* | 99 |
| *orf13* | 100 |
| *orf14* | 100 |
| *orf15* | 100 |
| *orf16* | 100 |
| *orf17* | 100 |
| *dam* | 99 |
| *orf19* | 99 |
| *orf20* | 100 |
| *intP* | 100 |
| *tcpA* | 99 |
| *tcpC* | 100 |
| *tcpD* | 100 |
| *tcpE* | 100 |
| *tcpF* | 99 |
| *tcpG* | 97 |
| *ltrA* | 100 |
| *tcpH* | 99 |
| *tcpI* | 100 |
| *tcpJ* | 100 |
| *orf32* | 100 |
| *orf33* | 100 |
| *dcm* | 98 |
| *orf35* | 99 |
| *orf36* | 98 |
| *orf37* | 99 |
| *orf38* | 99 |
| *orf39* | 100 |
| *orf40* | 98 |
| *orf41* | 93 |
| *orf42* | 97 |
| *orf43* | 98 |
| *orf44* | 99 |
| *orf45* | 100 |
| *orf46* | 100 |
| *orf47* | 100 |
| *orf48* | 100 |
| *orf49* | 100 |
| *orf50* | 99 |
| *orf51* | 100 |
| *orf52* | 99 |
| *orf53* | 97 |
| *orf54* | 99 |
| *orf55* | 100 |
| *orf56* | 100 |
| *orf57* | 100 |
| *orf58* | 94 |
| *orf59* | 100 |
| *orf60* | 100 |
| *orf61* | 100 |
| *orf62* | 100 |
| *orf63* | 100 |
| *orf64* | 100 |
| *orf65* | 100 |
| *orf66* | 100 |
| *orf67* | 99 |
| *orf68* | 100 |
| *orf69* | 100 |
| *orf70* | 99 |
| *orf71* | 100 |
| *orf72* | 100 |
| *orf73* | 100 |
| *net B* | 100 |
| *orf75* | 100 |
| *orf76* | 100 |
| *orf77* | 99 |
| *orf78* | 99 |
| *orf79* | 99 |
| *orf81* | 100 |
| *orf82* | 99 |

**Table S2 (B). Comparison of open reading frames pCpb2-CP1 and pJIR3844 NE *C. perfringens* plasmids by means of BLASTn analyses.** Open reading frames are labeled according to the annotation of plasmid pCpb2-CP1

| **CDS/ORF** | **% Identity** |
| --- | --- |
| *orf1* | 99 |
| *regB* | 97 |
| *orf3* | 100 |
| *orf4* | 100 |
| *orf5* | 100 |
| *rep* | 100 |
| *regCC* | 98 |
| *regD* | 99 |
| *orf9* | 99 |
| *orf10* | 100 |
| *orf11* | 100 |
| *cna* | 99 |
| *orf13* | 100 |
| *orf14* | 67 |
| *orf15* | 100 |
| *orf16* | 100 |
| *orf17* | 100 |
| *dam* | 99 |
| *orf19* | 100 |
| *orf20* | 100 |
| *intP* | 99 |
| *orf22* | 100 |
| *tcpA* | 100 |
| *tcpB* | 100 |
| *tcpC* | 99 |
| *tcpD* | 97 |
| *tcpE* | 94 |
| *tcpF’* | 95 |
| *tcpF* | 95 |
| *tcpG* | 97 |
| *ltrA* | 100 |
| *tcpH* | 95 |
| *tcpI* | 99 |
| *tcpJ* | 87 |
| *orf36* | 91 |
| *dcm* | 98 |
| *orf38* | 100 |
| *orf39* | 100 |
| *orf40* | 99 |
| *orf41* | 91 |
| *orf42* | 95 |
| *orf43* | 98 |
| *orf44* | 99 |
| *orf45* | 100 |
| *orf46* | 99 |
| *orf47* | 99 |
| *orf48* | 100 |
| *orf49* | 100 |
| *orf50* | 99 |
| *orf51* | 100 |
| *orf52* | 99 |
| *orf53* | 99 |
| *orf54* | 100 |
| *orf55* | 99 |
| *orf56* | 99 |
| *orf57* | 100 |
| *orf58* | 100 |
| *orf59* | 99 |
| *orf60* | 99 |
| *orf61* | 98 |
| *orf62* | 97 |
| *orf63* | 95 |
| *orf66* | 99 |
| *orf67* | 99 |
| *cpb2’* | 100 |
| *‘cpb2* | 100 |
| *orf72* | 100 |
| *orf73* | 100 |
